# Deciphering the Genetic Basis of Degenerative and Developmental Eye Disorders in 50 Pakistani Consanguineous Families Using Whole-Exome Sequencing

**DOI:** 10.3390/ijms26062715

**Published:** 2025-03-18

**Authors:** Ainee Zafar, Ruqia Mehmood Baig, Abida Arshad, Abdur Rashid, Sergey Oreshkov, Helen Nabiryo Frederiksen, Muhammad Ansar

**Affiliations:** 1 Department of Zoology, Wildlife and Fisheries, PMAS-Arid Agriculture University Rawalpindi, Rawalpindi 46000, Pakistan; ainee.jamal15@yahoo.com (A.Z.);; 2 Department of Ophthalmology, University of Lausanne, Jules Gonin Eye Hospital, Fondation Asile Des Aveugles, 1004 Lausanne, Switzerland; rashid.abdur@fa2.ch (A.R.); sergey.oreshkov@fa2.ch (S.O.); helen.frederiksen@fa2.ch (H.N.F.); 3 Advanced Molecular Genetics and Genomics Disease Research and Treatment Centre, Dow University of Health Sciences, Karachi 74200, Pakistan

**Keywords:** degenerative and developmental eye disorder, IRDs, anophthalmia, WES, autosomal recessive, Pakistani population, genetic analysis

## Abstract

Degenerative and developmental eye disorders, including inherited retinal dystrophies (IRDs), anophthalmia, and congenital cataracts arise from genetic mutations, causing progressive vision loss or congenital structural abnormalities. IRDs include a group of rare, genetically, and clinically heterogeneous retinal diseases. It is caused by variations in at least 324 genes, affecting numerous retinal regions. In addition to IRDs, other developmental eye disorders such as anophthalmia and congenital cataracts also have a strong genetic basis. Autosomal recessive IRDs, anophthalmia, and congenital cataracts are common in consanguineous populations. In many endogamous populations, including those in Pakistan, a significant proportion of IRD and anophthalmia cases remain genetically undiagnosed. The present study investigated the variations in IRDs, anophthalmia, and congenital cataracts genes in 50 affected families. These unrelated consanguineous families were recruited from the different provinces of Pakistan including Punjab, Khyber Pakhtoon Khwa, Sindh, Gilgit Baltistan, and Azad Kashmir. Whole exome sequencing (WES) was conducted for the proband of each family. An in-house customized pipeline examined the data, and bioinformatics analysis predicted the pathogenic effects of identified variants. The relevant identified DNA variants of selected families were assessed in parents and healthy siblings via Sanger sequencing. WES identified 12 novel variants across 10 known IRD-associated genes. The four most frequently implicated genes were *CRB1* (14.3%), *GUCY2D* (9.5%), *AIPL1* (9.5%), and *CERKL* (7.1%) that together accounted for 40.4% of all molecularly diagnosed cases. Additionally, 25 reported variants in 19 known IRDs, anophthalmia, and congenital cataracts-associated genes were found. Among the identified variants, p. Trp278X, a stop–gain mutation in the *AIPL1* (NM_014336) gene, was the most common causative variant detected. The most frequently observed phenotype was retinitis pigmentosa (46.5%) followed by Leber congenital amaurosis (18.6%). Furthermore, 98% of pedigrees (49 out of 50) were affected by autosomal recessive IRDs, anophthalmia and congenital cataracts. The discovery of 12 novel likely pathogenic variants in 10 IRD genes, 25 reported variants in 19 known IRDs, anophthalmia and congenital cataracts genes, atypical phenotypes, and inter and intra-familial variability underscores the genetic and phenotypic heterogeneity of developmental and degenerative eye disorders in the Pakistani population and further expands the mutational spectrum of genes associated with these ocular disorders.

## 1. Introduction

Degenerative and developmental eye disorders including IRDs, anophthalmia and congenital cataracts encompass a diverse group of conditions caused by genetic disruptions, leading to progressive vision loss or structural abnormalities from birth [1]. IRDs are a genetically diverse group of disorders that collectively affect around 1 in 2000 to 4000 people worldwide [2]. These conditions follow various inheritance patterns such as autosomal dominant, autosomal recessive, X-linked, and mitochondrial transmission. The genetic complexity of IRDs arises from mutations in approximately 324 genes (RetNet, https://RetNet.org/; accessed on 22 February 2025) involved in critical retinal functions, including visual cycle, photo-transduction cascade, ciliary structure, transport, outer segment structure, retinal development, metabolism, homeostasis, RNA splicing and gene transcription [3]. These mutations contribute to significant genetic and clinical heterogeneity, often resulting in variable disease severity and phenotypic expression, even among individuals within the same family. The complexity of IRDs is further influenced by allelic heterogeneity, genotypic variability, and epistatic interactions. IRDs are classically monogenic/Mendelian disorders such as retinitis pigmentosa (RP). This is in contrast to a complex, multifactorial disease such as age-related macular degeneration, which is influenced by multiple genetic and environmental factors, with age being the primary risk factor [4]. Rarely, mitochondrial DNA mutations also contribute to retinal degeneration, as seen in Leber hereditary optic neuropathy. IRDs can manifest as either ophthalmic or systemic disorders. Ophthalmic IRDs primarily affect vision and include RP, Stargardt disease (STGD), and cone–rod dystrophy (CRD). In contrast, systemic IRDs are associated with additional syndromes affecting other organs. Examples include Usher syndrome, Knobloch syndrome (KNO), Stickler syndrome (STL) and Bardet–Biedl syndrome [5]. IRDs often recur within families, and the recurrence risk for family members can often be predicted [6]. IRDs demonstrate both intra-familial and inter-familial phenotypic variability [7]. The clinical presentations of IRDs can be diverse, including retinal degeneration, cataracts, obesity, compromised immunity, and intellectual as well as speech disabilities [8]. Among developmental eye disorders, anophthalmia is a severe congenital eye disorder characterized by the complete absence of one or both eyes. It represents the most extreme phenotype within the anophthalmia–microphthalmia (A/M) spectrum, which also includes microphthalmia (small eye) and coloboma (ocular tissue defects). It results from disruptions in early ocular development with both monogenic and polygenic factors contributing to its genetic complexity. Variants in key developmental genes such as *SOX2, OTX2, FOXE3, RAX*, and *VSX2* have been implicated in the development of anophthalmia. Recessive anophthalmia is more common in consanguineous populations with mutations in *RAX, FOXE3*, and *ALDH1A3* associated with both isolated and syndromic forms. The global prevalence is estimated at 1 in 30,000 for anophthalmia, accounting for 3–12% of childhood visual impairment [9]. Similarly, among developmental eye disorders, congenital cataracts are a genetically diverse disorder causing lens opacity at birth, leading to visual impairment, with the highest incidence in Asia (7.43/10,000). While dominant forms are more common, recessive congenital cataract occurs in populations with high consanguinity, which is often linked to mutations in genes like *CRYBB3, FYCO1*, and *GJA8* [10]. Consanguinity significantly increases the risk of children acquiring congenital genetic diseases by expressing autosomal recessive genetic mutations inherited from their heterozygote carrier parents [11]. First-cousin marriage increases the risk of genetically-determined birth defects by 2–3% (i.e., 4–6% absolute risk) over the general population risk (2–3%). However, multiple generations of consanguinity may further increase this risk. In highly endogamous populations like Pakistan, the prevalence of rare Mendelian diseases is elevated, resulting in a higher incidence of autosomal recessive disorders [12]. Geographic constraints and a high prevalence of consanguineous marriages, with up to 80% occurring between first-degree relatives in some regions, contribute to the formation of genetic isolates carrying an increased frequency of disease-related founder variants. Furthermore, the absence of a proper database or case registration system has led to an incomplete understanding of congenital ocular defects and their prevalence in the country. Herein, we report the genetic findings from 50 consanguineous Pakistani pedigrees affected by IRDs, anophthalmia and congenital cataracts. Given the high rate of consanguinity in Pakistan, these families offer valuable insights into the genetic architecture of IRDs, particularly autosomal recessive forms. Through comprehensive genetic analysis by WES, this study aims to identify disease-causing variants, contributing to a deeper understanding of the molecular landscape of IRDs in the highly endogamous Pakistani population. Additionally, we explored inter- and intra-familial phenotypic heterogeneity, which sheds light on the variable clinical presentation of IRDs within Pakistani pedigrees. Understanding the specific frequency and spectrum of disease-associated gene variants across different regions of Pakistan is essential for developing effective genetic testing strategies and identifying population-specific variants [13,14]. Our findings not only expand the spectrum of pathogenic variants in known IRD, anophthalmia and congenital cataract-related genes but also emphasize the need for improved genetic screening and diagnostic resources in Pakistan.

## 2. Results

### 2.1. Pedigrees Analyzed

**Figure 1:**
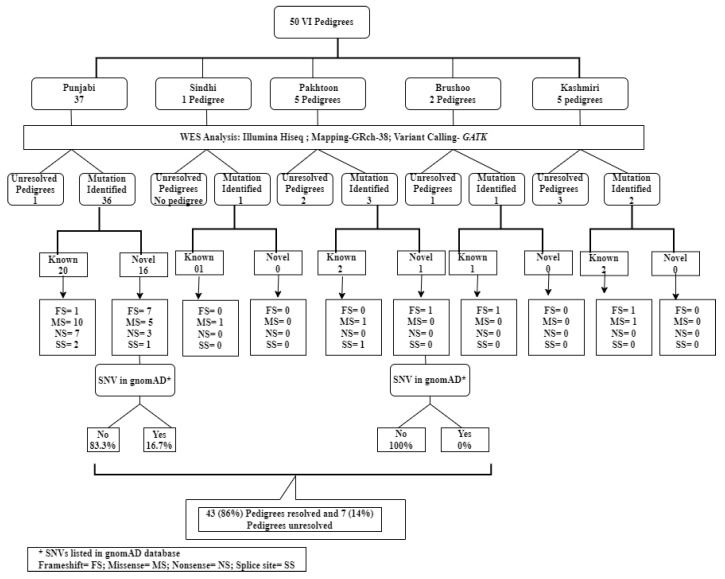
Summary of findings and population distribution of novel and known variations identified in various ethnic groups from Pakistan including Punjabi, Pakhtoon, Sindhi, Brushoo, and Kashmiri pedigrees.

**Figure 2:**
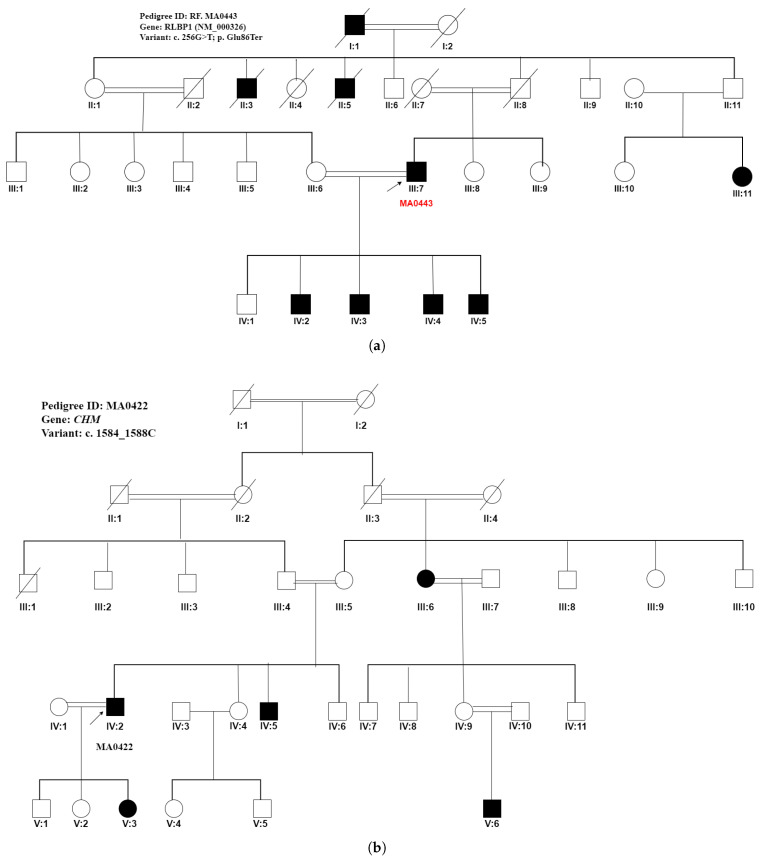
(**a**) A Punjabi pedigree with ID (RF. MA0443) initially classified as autosomal dominant. However, genetic analysis revealed that it followed a pseudo-dominant mode of inheritance of an autosomal recessive disease and was subsequently reclassified as autosomal recessive. WES identified a novel homozygous nonsense variant, c.256G>T (p.Glu86X), in exon 5 of the *RLBP1* gene in the proband. This variant was linked to autosomal recessive RP. (**b**) A Brushoo pedigree RF. MA0422 from Gilgit Baltistan, Province of Pakistan seggregating RP with an X-linked mode of inheritance. WES analysis identified an already reported homozygous variant c.1584_1587del, p.(Val529HisfsTer7) in the *CHM* (NM_000390) gene (MIM#300390), resulting in a truncation of the protein at the 3rd amino acid, seggregating with RP in this pedigree.

WES was performed on 50 probands from 50 unrelated Pakistani consanguineous pedigrees, initially diagnosed with IRDs, congenital cataracts, and structural anomalies. The pedigrees were from various provinces of Pakistan, including Punjab (37), Sindh (1), Khyber Pakhtunkhwa (5), Azad Kashmir (5), and Gilgit Baltistan (2) as shown in Figure 1. In this study, 86% of pedigrees (43/50) were genetically resolved by WES with 37 causal variants identified. The mode of inheritance was originally observed to be autosomal recessive in all pedigrees except one, which appeared to be autosomal dominant. Of the clinically diagnosed autosomal recessive pedigrees, 42 of 43 (97.6%) were confirmed to be autosomal recessive by genotyping. After WES analysis, the mode of inheritance was reclassified to two pedigrees from autosomal dominant to pseudodominant inheritance of autosomal recessive disease in pedigree (ID: RF. MA0443) and from autosomal recessive to X-linked due to a mutation in the X-linked gene, *CHM* in pedigree (ID: RF. MA0422), as shown in Figure 2, respectively.

### 2.2. Reclassification of Presenting Clinical Phenotypes Based on WES Analysis Results

**Figure 3:**
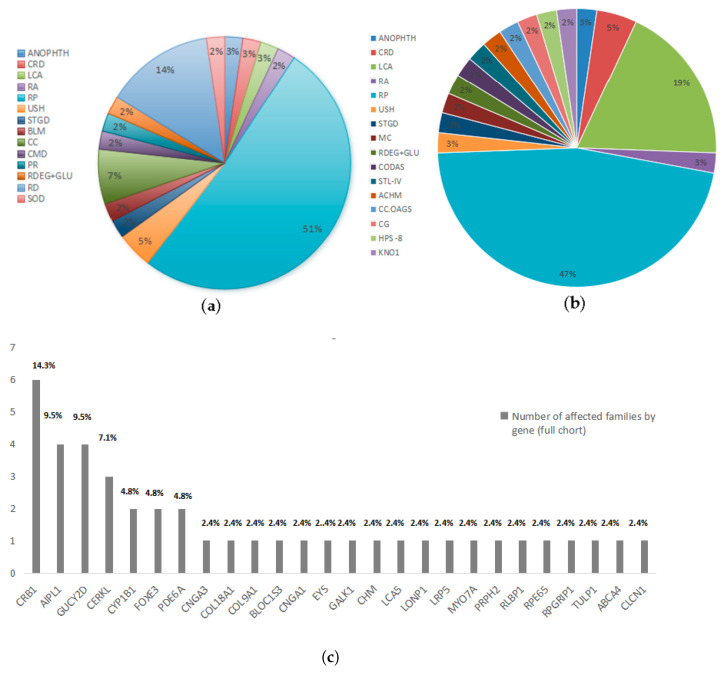
(**a**,**b**) A comparison diagram to illustrate the differences between initial clinical diagnosis and diagnosis after genetic testing (Retinitis Pigmentosa = RP; Leber congenital amaurosis = LCA; Retinal dystrophy = RD; Cone rod dystrophy = CRD; Myotonia congenita = MC; Retinal atrophy = RA; Pigmentary retinopathy = PR; Stickler syndrome type-IV = STL-IV; Stargardt = STGD; Bilateral maculopathy = BLM; Achromatopsia = ACHM; Cerebral, Ocular, Dental, Auricular, and Skeletal anomalies syndrome = CODAS syndrome; Congenital glaucoma = CG; Congenital macular degeneration = CMD; Usher syndrome= USH; Hermansky-Pudlak Syndrome 8 = HPS8; Anophthalmia = ANOPHTH; Congenital cataract = CC; Cataract-open angle glaucoma syndrome = CC.OAGS; Retinal degeneration plus glaucoma = RDEG+GLU; Congenital open angle glaucoma = C.OAG; Septo-optic dysplasia = SOD). (**c**) Spectrum of IRD Genes Identified in the Pakistani Population. The X-axis represents the identified IRD genes from the current study, while the Y-axis shows the number of pedigrees harboring mutations in these genes. In our consanguineous AR population, the top four most frequently implicated genes were *CRB1* (14.3%), *GUCY2D* (9.5%), *AIPL1* (9.5%), and *CERKL* (7.1%).

The initial clinical investigation of autosomal recessive pedigrees covered a wide range of phenotypic possibilities. Revisiting the clinical findings following WES analysis results led to the reclassification of clinical phenotypes in our pedigrees Figure 3 and Table 1. Six distinct phenotypes were identified in 13 pedigrees harboring novel IRD gene variants based on WES findings, including RP in five pedigrees, Leber congenital amaurosis in three pedigrees, cone-rod dystrophy in two pedigrees, and achromatopsia, Knobloch syndrome and Stickler syndrome type-IV in one pedigree each. Similarly, 12 distinct phenotypes were identified across 30 pedigrees harboring previously reported variants based on WES findings including RP in 15 pedigrees, LCA in 5 pedigrees, and congenital glaucoma, retinal atrophy, cerebral, ocular, dental, auricular, and skeletal anomalies syndrome (CODAS), Hermansky Pudlak syndrome 8, myotonia congenita, retinal degeneration plus glucoma, Stargadt disease, Usher syndrome, congenital cataracts and anophthalmia in one pedigree each. The phenotypes of seventeen families were reclassified based on genetic findings, as shown in Table 1.

### 2.3. Previously Reported Variants Identified in Known IRD Genes

**Figure 4:**
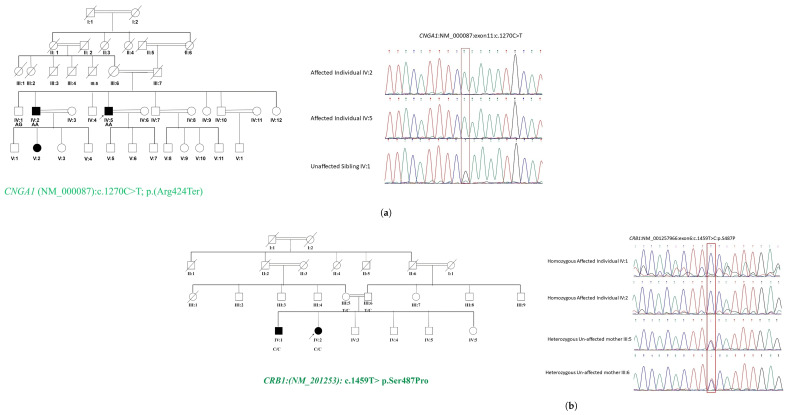
(**a**) The pedigree RF.MA0334 represents a Punjabi RP manifesting family harboring a novel nonsense stop-gain variant c.1270C>T; p.(Arg424Ter) in exon 10 of the *CNGA1* gene. The variant results in a premature stop codon at position 424 (Arg424Ter), leading to a truncated, non-functional protein. The Sanger sequencing confirmed that affected individuals (IV:2 and IV:5) were homozygous for the c.1270C>T variant and the normal sibling (IV:1) was heterozygous for the variant. (**b**) Kashmiri pedigree RF.MA0406 segregating RP in an autosomal recessive manner harbored a previously reported non-synonymous single nucleotide variant c.1459T>C; p. (Ser487Pro) in the *CRB1* gene (NM_201253). The Sanger sequencing confirmed that both affected individuals were homozygous for the c.1459T>C variant, while the parents were heterozygous for the same variant.

In our study, we identified 25 previously reported variants in 19 known genes in pedigrees detailed in Figure 4, Figures S1–S3 and Table S2 in Supplementary Material. These large consanguineous pedigrees included 24 Punjabi families, 1 Sindhi family, 2 Pakhtoon families, 2 pedigrees of Kashmiri origin, and one of Brushoo origin Figure 1. Overall, 24 variants were homozygous while 1 variant was an X-linked variant in pedigree-bearing RF. MA0422 Figure 2 as shown in Table S2 in Supplementary Material and Table 2. Genetic analysis revealed that *CRB1* variants were the most common cause of IRDs in six unrelated autosomal recessive consanguineous pedigrees. All six variants including chr1:197328458C>G, chr1:197442249G>C, chr1: 197328952T>C, chr1: 197435205-197435215del (identified in two unrelated pedigrees) and chr1:197421287T>C had been previously reported. In pedigree RF. MA0499 (Figure S1) with a presenting phenotype of retinal degeneration and glucoma, the *CYP1B1* (NM_000104) gene variant c.1310C>T, p. (Pro437Leu) shows variable expressivity. The male patient (IV:6) has glaucoma with progressive visual impairment leading to blindness. He also has nystagmus, retinal degeneration, and photophobia, indicating severe ocular involvement. In contrast, in the second affected female individual, the proband (IV:5) does not have glaucoma but experiences severe visual impairment, photophobia, retinal degeneration and cataracts, which may contribute to reduced vision.The patient (IV:5) was clinically examined at 2 years of age and reported symptoms of low vision and congenital photophobia consistent with the retinal degeneration phenotype. The age of onset of retinal degeneration in both affected (IV:5 and IV:6) members was during very early childhood, ranging from birth to 2 years of age. However, the fundus images of both patients at younger ages were not available. This phenotypic variability underscores the complexity of *CYP1B1*-related disease, suggesting the potential influence of genetic modifiers or environmental factors. The *CYP1B1* c.1310C>T variant in pedigree RF.MA0499 may be associated with both glaucoma and retinal degeneration, suggesting a broader ocular phenotype. Furthermore, the phenotype observed in patients IV:5 and IV:6 was also consistent with LCA, which is characterized by early-onset severe visual impairment, congenital photophobia, and retinal degeneration.

Considering the variability of IRDs and the potential for genetic heterogeneity, the possibility of an additional monogenic cause resembling LCA could not be ruled out especially in the context of a consanguineous pedigree. Bio-informatics analysis revealed that the proline located in the cytochrome P450 domain is essential for binding other molecules and interacts with residues in a domain also important for binding. The mutation might disrupt the interaction between these two domains, potentially affecting the protein’s function. Additionally, since proline is situated on the surface of the protein, its mutation could interfere with interactions with other molecules or different parts of the protein.

### 2.4. Novel Potentially Causative Variants Detected in Known IRD Genes

In 13 pedigrees as shown in Figure 2 and Figures S4–S8 in the Supplementary Material including 12 Punjabi and 1 Pakhtoon pedigrees, 12 novel homozygous variants in 10 different already reported IRDs related genes were detected (Table S2 in Supplementary Material).

#### 2.4.1. Non-Sense Mutations Observed in Unrelated IRD Pedigrees

Genetic analysis of a Punjabi-based pedigree RF. MA0334, as shown in Figure 4, including two affected patients and one unaffected individual, identified a novel nonsense stop-gain variant c.1270C>T; p.(Arg424Ter) in *CNGA1* (NM_000087). This variant, located in exon 10, was found to be associated with autosomal recessive RP, as shown in Figure 4.

The clinical phenotype observed in both patients is consistent with *CNGA1*-related RP with the presenting classic triad of RP including vessel attenuation, bony spicule pigmentation, and waxy pallor optic disc.

Both patients initially presented with night blindness. The proband (IV:5), aged 62, exhibited congenital onset of night blindness and photophobia, whereas the second patient (IV:2) developed night blindness at 60 years of age, demonstrating intra-familial variability. In the proband (IV:5), RP had an early onset with a remarkably slow progression, resulting in moderate visual impairment even after six decades. Conversely, patient (IV:2) experienced a late onset at 60 years, followed by rapid disease progression, leading to loss of color perception and complete blindness by 70 years. Partial blindness occurred in patient (IV:2) within five years of onset of symptoms at approximately 65 years. Sanger sequencing data analysis of the proband, affected individual, and unaffected sibling verified and recognized the given family structure and genetic relatedness. Evaluation of the Sanger sequencing data of patients and healthy siblings confirmed the shared biallelic loss of function variants in both affected individuals and the absence of the novel c. 1270C>T, p.(Arg424X) in the *CNGA1* gene in the unaffected sibling, suggesting this nonsense variant as the likely underlying cause of RP in these patients.

#### 2.4.2. Novel Frameshift Variants Detected in Known IRD Genes

**Figure 5:**
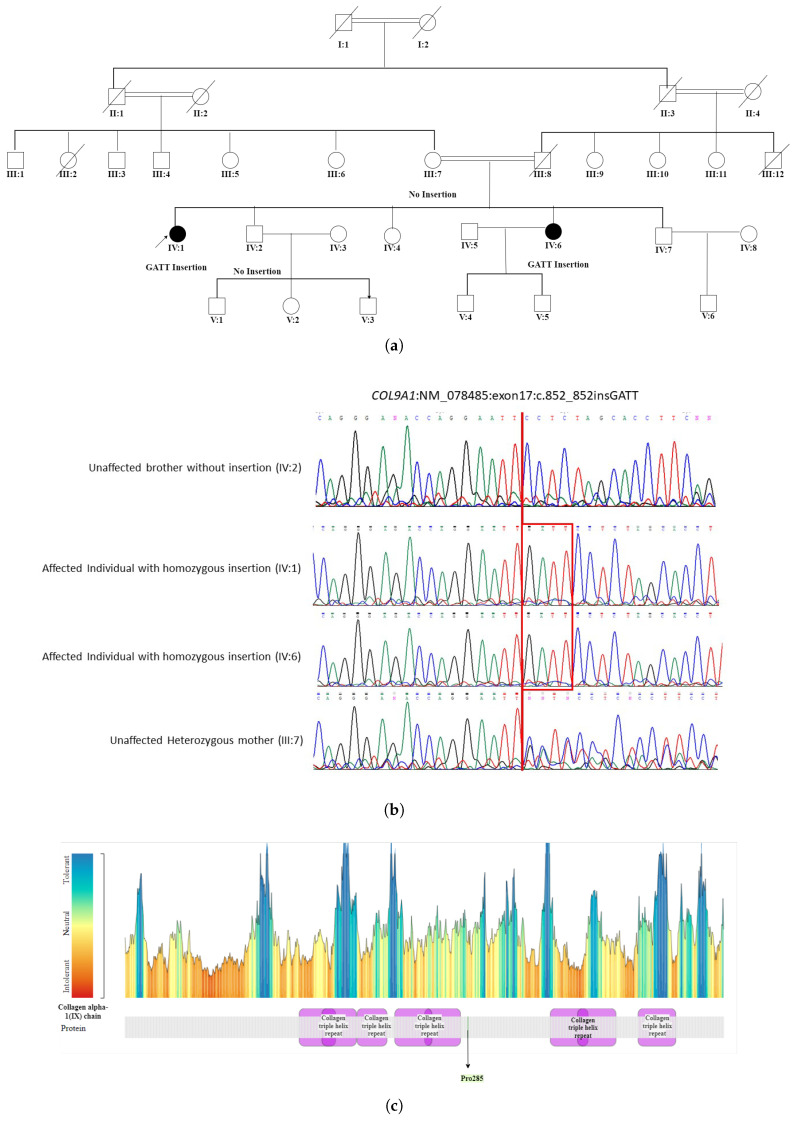
(**a**) WES in patient IV:1 (the proband) of a five-generation pedigree RF.MA0537 seggregating Stickler syndrome type 4 identified a novel homozygous nonsense mutation c.851_852insCAAT in the *COL9A1* gene, leading to the protein change p. (Pro285AsnfsTer20). (**b**) shows the Sanger sequencing results of family ID RF. MA0537. The affected individuals harbored homozygous insertion GATT. The mother was heterozygous while the unaffected brother lacked GATT insertion. (**c**) MetaDome health map represents STL-IV/COL9A1 residues.

1. A 20 bps homozygous frameshift deletion in *COL18A1* (NM_030582.4):c.3559_3577del p.(Ser1187AlafsTer18) was detected in a large Punjabi consanguineous pedigree Ref. MA0468 Figure S6. This homozygous deletion included 20 bps of exon 36. The deletion of the 20 bps sequence in exon 36 of *COL18A1* might produce a truncated and consequently non-functional protein owing to the frameshift of the coding region. PCR amplification and Sanger sequencing of the deleted region using primers located in the flanking sequence identified these specific boundaries of the frame shift deletion and its further segregation with Knobloch syndrome in the RF.MA0468 pedigree. The first affected individual presented with microphthalmia, early childhood photophobia, congenital cataract, severe visual impairment with light perception, bilateral retinal detachment, and chorioretinal atrophy. The second affected individual exhibited congenital strabismus, early childhood photophobia, corneal opacity, congenital cataract, unilateral retinal detachment, and chorioretinal atrophy. Notably, night blindness was absent in both individuals. While some of these features (e.g., retinal detachment, chorioretinal atrophy) are consistent with Knobloch syndrome, others are more unusual (e.g., microphthalmia, lack of nyctalopia) but may represent the phenotypic heterogeneity of this condition. MetaDome analysis revealed that the truncation is in a mildly intolerant region 20 residues before the endostatin domain. This deletion likely leads to the loss of the endostatin domain in collagen type XVIII alpha chain, which is associated with Knobloch syndrome. As endostatin binds zinc near its N-terminus, the mutation may impact the structure rather than the function [15].
2. The WES in patient IV:1 (proband) of a five-generation pedigree RF. MA0537 Figure 5 identified a novel homozygous nonsense mutation c.851_852insCAAT, p. Pro285AsnfsTer20 in the *COL9A1* gene. This novel sequence change led to a premature stop signal p. Pro285AsnfsTer20 in the *COL9A1* gene. This resulted in an expected disrupted or absent protein product. The family was initially suspected to have Usher syndrome due to hearing loss in both affected individuals. However, based on WES findings, it was reclassified as STL-IV. The affected members included two female patients, IV:1 (49 years) and IV:6 (35 years), who were born to first-degree relatives. Patient (IV:1) experienced severe visual impairment, including night and day blindness, deafness, and speech impairment, progressing to total blindness by age 40. In contrast, patient (IV:6) had milder symptoms with progressive vision loss, night blindness, cataracts, and hearing loss while retaining color and light perception. Neither patient exhibited facial dysmorphism. The reclassification to Stickler syndrome type 4 (STL-IV) aligns with key phenotypic features, including progressive vision loss, hearing impairment, and cataracts. In patient IV:1, night blindness occurred secondary to retinal detachment, which is a known complication of Stickler syndrome. In contrast, patient IV:6 exhibited night blindness while retaining light and color perception, suggesting milder retinal involvement or a distinct disease mechanism. Additionally, the absence of facial dysmorphism in both patients is consistent with STL-IV, which is typically associated with a non-syndromic presentation compared to other Stickler subtypes.

#### 2.4.3. Novel Missense Variants in Known IRD Genes

1. A novel *CNGA3* (NM_001298) variant, c.1774C>G, p. Pro592Ala, seggregated with the achromatopsia phenotype in the proband (IV:2) of a Punjabi family RF. MA0425, as seen in Figure S5. Both affected males were initially diagnosed with RP based on clinical evaluation. Their phenotype, characterized by photophobia, congenital nystagmus, and moderate visual impairment, was consistent with *CNGA3*-related achromatopsia.HOPE analysis showed that the wild-type residue proline is located in the cyclic nucleotide-binding domain. There are six invariant amino acids in this domain, three of which are glycine residues that are thought to be vital for maintaining the structural integrity of the beta barrel. The c.1774C>G substitution may affect the local structure by disrupting proline-induced special backbone conformation and impair binding site properties.
2. In the RF.MA0449 Punjabi pedigree (Figure S7) which included two affected members, WES identified a pathogenic novel homozygous variant c.1171T>C, p.(Cys391Arg) in exon 4 of the *GUCY2D* (NM_000180) gene, which was initially associated with bilateral maculopathy. However, due to the presence of congenital nystagmus, early-onset blindness, bilateral maculopathy, and photophobia in both affected individuals, the condition was reclassified as LCA. Bioinformatics analysis revealed a difference in charge between cysteine (neutral) and arginine (positive) residues. Cysteine is located in the receptor–ligand binding region, and the c.1171T>C variation introduces a charge that may generate the repulsion of ligands or other nearby residues bearing the same charge.
3. Analysis of a Punjabi pedigree RF. MA0454 Figure S8 with three affected members with the proband (IV:3) available for the WES study detected a homozygous potentially pathogenic novel variant c.626T>G, p. (Val209Gly) in the *PRPH2* gene linked with the autosomal recessive LCA phenotype. The clinical changes in the affected individuals including nystagmus, mottled fovea, macular atrophy, and congenital photophobia are consistent with the phenotype of autosomal recessive LCA. Bioinformatics analysis revealed that the variation is located within a stretch of residues, namely lumenal repeats, that are repeated in the peripherin/rom-1 protein. The substitution replaces valine with glycine at position 209. Glycine’s high flexibility could disrupt the rigidity of the specific repeat in the protein structure. Since valine is located near a highly conserved position, this substitution with glycine can lead to a loss of function in the protein.

## 3. Discussion

The analysis of the entire exome sequence of the present cohort consisted of 50 probands from 50 consanguineous Pakistani unrelated autosomal recessive pedigrees manifesting degenerative (IRDs) (48 pedigrees) and developmental eye disorders including anophthalmia and congenital cataracts (1 pedigree each). WES detected 37 causal variants in 43/50 (86%) pedigrees, as shown in Table 3. Among the 37 causative pathogenic variants identified, 12 (32.4%) were novel, and 25 (67.6%) were already reported variants in known IRDs, anophthalmia and congenital cataracts-associated genes. WES findings identified variations in 26 known IRD genes with success, as shown in Figure 3.

The remaining seven pedigrees underwent screening for variations in known IRD genes without success. However, variants in non-coding regions of well-established known IRD genes or novel genes with unidentified impact or yet to be explained/annotated may certainly contribute to the phenotype in the remaining six unresolved pedigrees. In pedigree RF. MA0443 shown in Figure 2, *RLBP1* variant c.256G>T, p. Glu86X led to a rare pseudo-dominant inheritance pattern, as the proband, who is homozygous for the mutation, has a partner with a heterozygous mutation. In this case, there is no horizontal transmission pattern on the pedigree, and patients can be seen across all four generations [16]. Furthermore, the carrier parent (III:6) had affected children with the affected person (III:7). Consanguinity in this family, which is affected by an autosomal recessive disorder, has resulted in a vertical transmission pattern of arRP on the pedigree [17]. This case broadens our understanding of genetic analysis in families manifesting apparent autosomal dominant RP, highlighting the impact of consanguinity and the need to consider pseudo-dominant patterns in genetic evaluations. In another pedigree, an 11 bps frameshift deletion c.3007_ 3016del of *CRB1* (NM_001193640.2) gene seggregated with the two different phenotypes, RP and LCA, in two unrelated consanguineous families, RF. MA0341and RF.MA0503, respectively. Both large families originated from the Punjab province of Pakistan and have Punjabi ethnicity. The association of the same variant c.3007_ 3016del with both RP and LCA in different families might be influenced by genetic modifiers, residual protein activity, and differences in retinal development. Further variation in phenotype may be contributed by variable expressivity and environmental factors. Generally, novel variations were discovered in known IRD genes in 13 pedigrees, while previously documented variations were identified in 30 autosomal recessive IRD, anophthalmia and congenital cataracts manifesting pedigrees. The majority of the variations were missense mutations (18). There were 10 stop–gain variants, 7 frameshift deletions, and 4 frameshift insertions, while only 4 were pathogenic splice-altering variants. Overall, 17 distinct phenotypes were identified during the study. Atypical phenotypes were identified in 4 out of 43 autosomal recessive large IRD and congenital cataracts manifesting pedigrees (9.3%). Intra-familial phenotypic heterogeneity was observed in 10 pedigrees. The phenotypes of 17 families were reclassified based on WES findings. The initial clinical diagnosis for three pedigrees was congenital cataracts. However, a reassessment of clinical findings following WES analysis led to the reclassification of their clinical phenotypes as congenital glaucoma, cone–rod dystrophy, and CODAS syndrome, respectively. This underscores the crucial role of genetic testing in improving diagnostic accuracy and guiding more precise clinical management. Interestingly, approximately 32.4% of the novel causative variants of known genes out of the total variants identified from our consanguineous pedigrees point to the genetic heterogeneity of IRDs in understudied Pakistani populations. The genetic landscape of IRDs exhibits notable differences between populations, as evidenced by studies from the United Kingdom and Pakistan. In a UK cohort of over 3000 families, Pontikos et al. [18] identified the top twenty implicated genes with the five most prevalent being *ABCA4* (20.8%), *USH2A* (9.1%), *RPGR* (5.1%), *PRPH2* (4.6%), and *BEST1* (3.9%). Collectively, these five genes accounted for a substantial proportion of molecularly diagnosed families. Overall, the top 20 implicated genes contributed to 71.8% of all molecularly diagnosed cases. In contrast, research focusing on Pakistani populations has highlighted a different distribution of prevalent IRD genes. A systematic review of IRDs in Pakistan reported that mutations in the *PDE6A* (5.32%) gene were the leading cause, which were followed by mutations in the *TULP1* (4.76%) gene [19]. Additionally, a study exploring the genetic landscape of retinal diseases in northwestern Pakistan identified prevalent mutations in genes such as *ABCA4, BBS2* and *CNGA1* as the most frequently associated IRD genes [20]. In contrast, the present study focusing on 48 Pakistani IRD families identified the most frequently implicated genes to be *CRB1* (14.3%), *AIPL1* (9.5%), *GUCY2D* (9.5%) and *CERKL* (7.1%), which together accounted for 40.4% of all molecularly diagnosed cases. This concurrence underscores the importance of these genes in retinal dystrophies and suggests a potential focus for future genetic screening and therapeutic interventions. Notably, our findings align with the recent study [21], which demonstrated that young children with *AIPL1*-related retinal dystrophy benefited substantially from the subretinal administration of rAAV8.hRKp.AIPL1 with improved visual acuity, functional vision, and evidence of some protection against progressive retinal degeneration without serious adverse effects. While *ABCA4* mutations are a common cause of IRDs in both UK and northwestern Pakistan regions, their prevalence in the UK is linked to a broader carrier frequency of 1 in 20 in certain populations, whereas in northwestern Pakistan, it is primarily due to a specific founder mutation amplified by consanguinity and genetic isolation. Notably, *ABCA4*, the most prevalent gene in the UK cohort, was not among the top genes in the present study. These disparities may be attributed to factors such as high consanguinity rates in Pakistan, leading to a higher prevalence of autosomal recessive disorders, and the presence of population-specific founder mutations. These findings provide critical evidence for the interpretation of novel variants in known IRD-associated genes, particularly in underrepresented, multiplex, unexplored non-European populations like the Pakistani population. An exponential surge in the discovery of novel syndromic and non-syndromic IRD genes ensued in two major periods: 2000– 2005 and 2010–2015, respectively [22]. These two spurts coincided with the prime expansion and development of cutting-edge genome analysis tools that further broadened our understanding of genomic architecture. Recent studies also reveal the role of atypical phenotypic and genomic alterations in IRD genes in disease pathology [23,24,25]. Our results are compatible with the general observation that the detection of novel syndromic and non-syndromic IRD genes has approached a plateau phase, and atypical phenotypic and genomic changes in known IRD genes may solve about 10 percent to 15 percent of unsolved cases [24]. The major population involved in our WES analysis is from the largest province of Pakistan, the Punjab. Heretofore, the genetic architecture of IRD in the Punjabi population was understudied and not well understood. The Pakistani population has a unique structure with predominant endogamous multiplex sub-populations and surging consanguinity in these sub-populations [26,27,28]. The earlier research studies on large recessive IRD manifesting pedigrees from Punjab [14,29] identified homozygous causative variants in about 149 families, which is almost 71%. Among the resolved Punjabi pedigrees, 97.5% had variations in already known IRD genes. These findings are parallel to our present study wherein 97.6% of variations are homozygous and are found in already-known IRD, anophthalmia, and congenital cataracts-related known genes. Among the variations identified in known IRD genes, the p. Trp278X stop-gain mutation in the *AIPL1* (NM_014336) gene is found to be the most common causative variant detected in four unrelated pedigrees in our study followed by the p. Arg257X stop–gain mutation in the *CERKL* (NM 201548) gene and c.650_651dup, which is a frameshift insertion in the *PDE6A* (NM_000440) gene identified in every two pedigrees, respectively. Furthermore, *CRB1* gene variants with known substitutions are the most frequent cause of RP in Pakistani patients followed by *GUCY2D* and *AIPL1* variants identified in four pedigrees each. Importantly, *CRB1* variants are known to be one of the commonest causes of autosomal recessive RP or early-onset retinal degeneration reported from different populations globally [30]. *CRB1* biallelic variants have been linked to a variety of inherited retinal degenerations including early-onset RP [31]. These findings are in contrast to previously conducted studies in the Pakistani population wherein the p.Pro363Thr in *RPE65* genes was found to be the most common causative variation found in the Punjabi population [29,32,33]. This variant was observed only in the South Asian population (gnomAD database). The two independent studies on a large cohort of autosomal recessive Pakistani pedigrees also asserted 70% novel variants and 30% already reported variants in IRD genes and causative variants in 60% of families with the detection of the same percentage of novel mutations in their study cohort, respectively [34,35]. Consistent with these results, the pathogenic/likely pathogenic variations detection rate was 43/50 (86%) in our cohort, and they were detected in 86% percent of the families. Additionally, only 34% of these identified causative mutations are listed in the gnomAD database. The novel causative variants identified in the Pakistani pedigrees either occur at very low frequency in the South Asian population or are absent, signifying those variants to be unique to the Pakistani population. Additionally, these were detected only in one or a few pedigrees despite the autozygous and endogamous nature of the Pakistani population, suggesting that the origin of those variants is either more recent or has arisen from the indigenous Pakistani population that might be poorly represented in gnomAD data. Overall, our research study highlights the phenotypic and genetic diversity of both developmental and degenerative eye disorders in the Pakistani population. Furthermore, the number of consanguineous families studied from each ethnic group including Punjabi, Sindhi, Pakhtoon, Brushoo, and Kashmiri is small. Analysis of further IRD, anophthalmia and congenital cataracts manifesting families from the understudied endogamous Pakistani populations may offer better and deeper insight into the molecular and genetic architecture of the same in the Pakistani population. The most common phenotype in the present cohort was RP, observed in 20 out of 43 cases (46.5%), followed by LCA in 8 out of 43 cases (18.6%). This distribution highlights RP as the predominant inherited retinal disorder in the Pakistani population.The present study also broadens the phenotypic spectrum of RP, LCA, CRD, HPS8, STL4, and USH in the Pakistani population. Furthermore, the identified variants were present in evolutionary highly conserved regions of the encoded proteins. Furthermore, 98% of pedigrees (49/50) were affected by autosomal recessive IRDs, anophthalmia and congenital cataracts. The global carrier rate for autosomal recessive inherited retinal diseases is estimated to be around 40% [18], indicating that a substantial portion of the population carries at least one mutation associated with these conditions. However, in Pakistan, the carrier rate is likely higher due to the widespread practice of consanguineous marriages, which accounts for over 70% of IRD cases. Pakistan has the highest rates of consanguinity in the world with nearly two thirds marrying cousins [36]. Consequently, more than 95% of reported IRDs in the country are recessively inherited, far exceeding global trends [19]. This is primarily driven by the inheritance of biallelic pathogenic mutations from both parents, which is a result of endogamy and reduced genetic diversity. The increased transmission of these mutations suggests a higher carrier frequency than the global average, underscoring the urgent need for genetic screening and awareness programs to reduce the prevalence and impact of inherited retinal diseases in Pakistan. The continuity of consanguineous marriages over generations upsurges/increases the risk of autosomal recessive disorders. It is important to note that this study does not propose changes to long-standing cultural practices, which hold significant societal value. Instead, the focus is on pre-conception carrier screening and prenatal testing as essential tools for informed decision making. Additionally, the significance of early diagnosis and access to effective support for children with heritable eye conditions is emphasized. With approximately 80% of consanguineous marriages in Pakistan occurring between first cousins, prenatal genetic diagnosis, genetic counseling, and future gene therapy for IRDs and structural anomalies are essential in reducing visual impairment in the Pakistani population. Furthermore, we hope that the correct categorization of the clinical relevance of numerous discovered novel pathogenic/likely pathogenic gene variants by using population-specific data and corresponding gene impact will definitely facilitate understanding of the molecular pathogenesis of developmental and degenerative eye disorders in the Pakistani population [14]. It will further help clinicians better understand IRDs, thus leading to an earlier and precise diagnosis and treatment of patients, since in the majority of IRDs, vision decreases gradually. An early and more correct diagnosis may provide patients sufficient time to at least adapt to these changes.

## 4. Materials and Methods

### 4.1. Identification of Consanguineous Families Manifesting Various Eye Disorders

Families with two or more visually impaired individuals were identified and visited at home or in the concerned hospitals. A questionnaire was used to collect clinical and medical information regarding consanguineous relationships between parents, risk factors, and family history. Multiple loops of consanguinity were accounted for in our pedigrees to ensure a comprehensive analysis of genetic inheritance. Color fundus photographs were taken when available. Due to limited resources, autofluorescence and OCT imaging were not used in this study. Photographs of affected individuals were taken with a digital camera to capture key features of patients with developmental abnormalities. The clinical examination of patients was conducted at nearby local government and private hospitals. The sample size comprised 50 unrelated consanguineous families each containing at least two visually affected members.

### 4.2. Inclusion Criteria

Patients had at least two affected individuals per family with considerable concordant phenotypes, either in the same sibship or in different branches (cousins) of the family. In the latter, evidence of the possible common origin of a pathogenic variant was sought. If genetic diagnosis was performed, the causative gene had not been identified. Any suspicion of non-genetic origin of the disease in under-study subjects was ruled out.

### 4.3. Phenotyping and Collection of Saliva

Before collecting saliva samples, all subjects were inspected by an authorized physician in the area, and detailed medical history forms were completed. Saliva samples from all available affected individuals, parents, and normal siblings were drawn using ORAGENE-DNA (OG-500) Saliva Kits. Manual extraction of saliva DNA for genetic analysis from saliva samples was carried out using the prepIT.L2P kit according to the manufacturer’s protocol.

### 4.4. Approval by Ethical Committee

The study was conducted with approval from the Bioethics Committee of PMAS-Arid Agriculture University, Rawalpindi. Written consents were obtained from either parents or legal representatives for the genetic analysis. Following the recommendations of the Ethical Committee, variants with potential diagnostic value (i.e., relevant to the phenotype in question) were reported to the patient’s referring physicians for genetic counseling, further diagnostics, and reproductive actions.

### 4.5. Whole Exome Sequencing

WES was conducted at Geneva University Hospital, Switzerland, using the SureSelect Human All Exon kit v5 (Agilent Technologies, Santa Clara, CA, USA) on an Illumina HiSeq4000 platform [37]. Exome data analysis followed a customized pipeline incorporating the Burrows–Wheeler Aligner (BWA) 0.7.18, SAMtools, PICARD 3.1.0 (http://broadinstitute.github.io/picard/, and GATK 4.5.0 [38]. Sequencing reads were aligned to the reference human genome GRCh38 using BWA with variant calling performed using GATK and SAMtools 1.19.2 [37,39]. WES was conducted on a single affected member per family, achieving an overall mean-depth base coverage of at least 173-fold, ensuring that exome regions were covered at a minimum depth of 20-fold. Initially, variants in genes associated with IRDs and structural anomalies were extracted from the WES variant files to identify potential genetic diagnoses. Variants with a minor allele frequency below 1% in GNOMAD v3.1 [40] were prioritized based on several criteria: (i) predicted deleteriousness scores from CADD PHRED v1.6 [41] and conservation scores from GERP++ [42], (ii) classification according to InterVar [43], based on ACMG variant scoring guidelines, which categorize variants as Benign, Likely Benign, Variant of Uncertain Significance (VUS), Likely Pathogenic, or Pathogenic. (iii), the severity of the genetic alteration (e.g., truncation, missense, or synonymous variants). (iv) splice site predictions using dbscSNV v1.1 [44]. In cases where WES identified likely pathogenic or pathogenic variants associated with IRDs or structural anomalies, but their correlation with the clinical phenotype remained uncertain, further validation was performed through Sanger sequencing. This involved genotyping all family members to assess segregation of the variants with disease phenotypes. Additionally, affected individuals, parents, and one healthy sibling from families MA0334, MA0362, MA0387, MA0406, MA0468, MA0477, MA0503, MA0510, and MA0537 underwent genotyping via Sanger sequencing for the identified variants [45].

### 4.6. Sanger Sequencing of Samples from Selected VI Families

For the validation and segregation analysis of potential variants, Sanger sequencing was performed. Sanger sequencing was performed at Microsynth, Balgach, Switzerland. The primers (Supplementary Table S1—Summary of primers) used to PCR amplify the particular locus were designed using the online interface/program of Primer 3plus https://www.bioinformatics.nl/cgi-bin/primer3plus/primer3plus.cgi accessed on 20 September 2023. http://bioinfo.ut.ee/primer3-0.4.0/ (Supplementary Table S1), the specificity of which was double-checked by an in silico PCR tool of UCSC genome browser https://genome.ucsc.edu/cgi-bin/hgPcr accessed on 20 September 2023. The sequencing reaction involved 9 selected affected families and 21 normal controls, utilizing the big dye terminator Sanger sequencing kit [46]. Following the reaction, samples were electrophoresed on a Genetic Analyzer 3130, and the resultant chromatograms were analyzed using ChromasPro version 3.

### 4.7. Bioinformatics Analysis for Protein Prediction

Computational analysis was conducted to predict the effects of likely pathogenic or pathogenic variants on encoded proteins. For non-synonymous substitutions, tools such as InterVar [43], CADD [41], and the HOPE (Have Your Protein Explained) protein prediction web tools were utilized. Frameshift variants were analyzed using MetaDome v1.0.1 [47].

## 5. Conclusions

The discovery of novel likely pathogenic variants in 10 genes, previously reported variants in 19 IRDs, anophthalmia and congenital cataract-associated genes, atypical phenotypes and inter and intra-familial variability confirms the genetic and phenotypic heterogeneity of IRDs in the Pakistani population and further expands the mutational spectrum of IRD genes. The atypical phenotypes and clinical heterogeneity among patients may be due to other factors affecting the phenotype, including variants in modifier genes.

## Figures and Tables

**Table 1:** Pedigree analysis, phenotypic assessment, and genetic findings.

Pedigree ID	Initial Phenotype	Re-Assessed Phenotype	Gene	Variant
MA0315	Retinal dystrophy	Leber congenital amaurosis	*LCA5*	c.1151del
			(NM_001122769)	p.(Pro384GlnfsTer18)
MA0383	Retinitis pigmentosa	Myotonia congenita	*CLCN1*	c.2647C>A
			(NM_000083)	p.(Pro883Thr)
MA0392	Septo-optic dysplasia	Leber congenital amaurosis	*AIPL1*	c.645G>A
			(NM_001033054.3)	p.(Trp215Ter)
MA0422	Pigmentary	Retinitis pigmentosa	*CHM*	c.1584_1587del
		retinopathy	(NM_000390)	p.(Val529HisfsTer7)
MA0425	Retinitis pigmentosa	Achromatopsia	*CNGA3*	c.1720C>G
			(NM_001079878)	p.Pro574Ala
MA0435	Stargardt disease	Leber congenital amaurosis	*AIPL1*	c.834G>A
			(NM_014336)	p.Trp278Ter
MA0449	Bilateral maculopathy	Leber congenital amaurosis	*GUCY2D*	c.1171T>C
			(NM_000180)	p.Cys391Arg
MA0454	Congenital macular	Leber congenital amaurosis	*PRPH2*	c.626T>G
			(NM_000322)	p.Val209Gly
MA0468	Retinal dystrophy	Knobloch syndrome	*COL18A1*	c.3559_3577del
			(NM_030582.4)	p.(Ser1187AlafsTer18)
MA0477	Retinal dystrophy	Hermansky–Pudlak	*BLOC1S3*	c.499C>T
		syndrome-8	(NM_212550)	p.(Leu167Phe)
MA0489	Retinal dystrophy	Congenital syndrome	*FOXE3*	c.720C>A
		cataract–glaucoma	(NM_012186)	p.(Cys240Ter)
MA0503	Retinal dystrophy	Leber congenital amaurosis	*CRB1*	c.3007_3016del
			(NM_001193640.2)	p.(Gly1003IlefsTer23)
MA0507	Retinal dystrophy	Leber congenital amaurosis	*AIPL1*	c.664T>C
			(NM_014336)	p.(Trp222Arg)
MA0510	Congenital cataract	Cone–rod dystrophy	*GUCY2D*	c.2965dup
			(NM_000180)	p.(Val989GlyfsTer83)
MA0515	Congenital cataract	Congenital glaucoma	*CYP1B1*	c.1063C>T
			(NM_000104)	p.(Arg355Ter)
MA0524	Congenital cataract	CODAS syndrome	*LONP1*	c.1448G>A
			(NM_001276480)	p. (Arg483His)
MA0537	Usher syndrome	Stickler syndrome type-IV	*COL9A1*	c.851_852insCAAT
			(NM_078485.4)	p.(Pro285AsnfsTer20)

**Table 2:** Clinical features of patients from pedigrees harboring genetic variants included in this study.

Family ID	Patient ID/Gender	Age at Enrollment (Years)	Initial Phenotype	Phenotype After WES	Initial Complaint with Age of Onset (Years)	No. of Affected Individuals	Clinical Symptoms	Identified Variant
MA0320	IV:2/Female (Proband)	27	ar RP	ar RP	By birth night blindness	02	Night blindness, photophobia, blindness since 13 years of age	*TULP1*(NM_001289395.2):c.417del p.(Lys140ArgfsTer2) Homozygous
	IV:3/Female	23	ar RP	ar RP	By birth night blindness	02	Night blindness, photophobia, blindness since 13 years of age	*TULP1*(NM_001289395.2):c.417del p.(Lys140ArgfsTer2) Homozygous
MA0324	IV:6/Male	31	ar RP	ar RP	Low vision and color blindness	02	Night blindness, photophobia, nystagmus, complete blindness at 14 years of age	*CRB1*(NM_001193640.2):c.107C>G p.(Ser36Ter) Homozygous
	IV:7/Female		ar RP	ar RP	Low vision	02	Night blindness, photophobia, nystagmus, complete blindness at 12 years of age	*CRB1*(NM_001193640.2):c.107C>G p.(Ser36Ter) Homozygous
MA0356	III:4/Male (Proband)	35	ar RP	ar RP	By birth night blindness	05	Night blindness, photophobia, complete blindness at 25 years of age	*CRB1*(NM_001193640.2):c.3626G>C p.(Cys1209Ser) Homozygous
	III:7/Male	44	ar RP	ar RP	By birth night blindness	05	Night blindness, photophobia, complete blindness at 25 years of age, corneal opacities	*CRB1*(NM_001193640.2):c.3626G>C p.(Cys1209Ser) Homozygous
MA0406	IV:1/Male	18	ar RP	ar RP	Low vision at 10 years	02	Night blindness, photophobia, low vision, slow progression of RP	*CRB1*(NM_001193640.2):c.1123T>C p.(Ser375Pro) Homozygous
	IV:2/Female (Proband)	32	ar RP	ar RP	Night blindness plus photophobia at 26 years	02	Night blindness, photophobia, low vision, slow progression of RP	*CRB1*(NM_001193640.2):c.1123T>C p.(Ser375Pro) Homozygous
MA0425	IV:2/Male (Proband)	06	arRP	ar achromatopsia	Photophobia, nystagmus, low vision	02	Night blindness, photophobia, nystagmus, low vision, microphthalmia	*CNGA3*(NM_001079878):c.1720C>G p.Pro574Ala Homozygous
	IV:1/Male	02	arRP	ar achromatopsia	Myopia since 4 months of age	02	Night blindness, photophobia, nystagmus, low vision	*CNGA3*(NM_001079878):c.1720C>G p.Pro574Ala Homozygous
MA0510	IV:8/Male (Proband)	36	Congenital cataract	Cone–rod dystrophy	By birth day blindness	02	Day blindness, night blindness, photophobia, nystagmus, complete blindness since 23 years of age	*GUCY2D*(NM_000180):c.2965dup p.(Val989GlyfsTer83) Homozygous
	IV:9/Male	27	Congenital cataract	Cone–rod dystrophy	By birth day blindness	02	Day blindness, photophobia, nystagmus, complete blindness since 23 years of age, bilateral cataracts	*GUCY2D*(NM_000180):c.2965dup p.(Val989GlyfsTer83) Homozygous
MA0337	IV:1/Male	36	arRP	arRP	Low vision since childhood	03	Night blindness, nystagmus, complete blindness at 25 years of age	*CERKL*(NM_001160277.2):c.715C>T, p.(Arg239Ter) Homozygous
	IV:3/Male (Proband)	40	arRP	arRP	Low vision since childhood	03	Night blindness, nystagmus, partial blindness	*CERKL*(NM_001160277.2):c.715C>T, p.(Arg239Ter) Homozygous
MA0371	IV:2/Female	27	arRP	arRP	Night blindness since 11 years	02	Night blindness, photophobia, complete blindness since 24 years of age, microphthalmia	*CERKL*(NM_001160277.2):c.715C>T, p.(Arg239Ter) Homozygous
	IV:4/Male (Proband)	34	arRP	arRP	Night blindness since 18 years	02	Night blindness, photophobia, complete blindness since 27 years of age	*CERKL*(NM_001160277.2):c.715C>T, p.(Arg239Ter) Homozygous
MA0380	III:1/Male (Proband)	34	arRP	arRP	Night blindness since 10 years	03	Night blindness, photophobia, severe visual impairment blindness since 29 years of age	*CRB1*(NM_001193640.2):c.601T>C, p. Cys210Arg Homozygous
	III:7/Male	37	arRP	arRP	By birth low vision	03	Night blindness, photophobia, nystagmus, severe visual impairment since 33 years of age	*CRB1*(NM_001193640.2):c.601T>C, p. Cys210Arg Homozygous
MA0400	IV:3/Male	23	arRP	arRP	Low vision since 7 years of age	02	Night blindness, photophobia, nystagmus, complete blindness in one eye since 19 years of age and cataracts	*LRP5*(NM_001291902.2):c. 430G>A, p.(Val144Ile) Homozygous
	IV:4/Male	27	arRP	arRP	Low vision since 7 years of age	02	Night blindness, photophobia, nystagmus, Complete blindness in one eye since 19 years of age and cataracts	*LRP5*(NM_001291902.2):c. 430G>A, p.(Val144Ile) Homozygous
MA0413	III:4/Female (Proband)	46	arRP	arRP	Low vision since 14 years of age	02	Night blindness, complete blindness since 17 years of age	*PDE6A*(NM_000440): c.650_651dup, p.(Ala218LeufsTer4) Homozygous
	III:7/Male	36	arRP	arRP	Night blindness since 4 years of age	02	Night blindness, photophobia, blindness since 4 years of age and cataracts	*PDE6A*(NM_000440): c.650_651dup, p.(Ala218LeufsTer4) Homozygous
MA0430	IV:4/Female (Proband)	12	arRP	arRP	Low vision since 3.5 years of age	02	Night blindness, blindness since 3.5 years of age and squint	*PDE6A*(NM_000440): c.650_651dup, p.(Ala218LeufsTer4) Homozygous
	IV:6/Male	06	arRP	arRP	Low vision since 2.5 years of age	02	Night blindness, blindness since 2.5 years of age and squint	*PDE6A*(NM_000440): c.650_651dup, p.(Ala218LeufsTer4) Homozygous
MA0341	III:1/Male	20	arRP	arRP	By birth low vision	02	By birth night blindness and photophobia, nystagmus, complete blindness at the age of 15 years, corneal opacities	*CRB1*(NM_001193640.2): c.3007_3016del, p.(Gly1003IlefsTer23) Homozygous
	III:4/Male (Proband)	22	arRP	arRP	Low vision at the age of 2 years	02	Night blindness and photophobia since childhood, nystagmus, blindness at the age of 15 years, corneal opacities	*CRB1*(NM_001193640.2): c.3007_3016del, p.(Gly1003IlefsTer23) Homozygous
MA0503	IV:1/Male (Proband)	43	arRD	arLCA	By birth blindness	02	By birth night blindness, by birth photophobia, complete blindness at 30 years of age, cataracts	*CRB1*(NM_001193640.2):c.3007_3016del, p.(Gly1003IlefsTer23) Homozygous
	IV:11/Male	46	arRD	arLCA	Very low vision since two years of age	02	By birth night blindness, by birth photophobia, complete blindness at 2 years of age	*CRB1*(NM_001193640.2):c.3007_3016del, p.(Gly1003IlefsTer23) Homozygous
MA0315	IV:3/Male	22	arRD	arLCA	By birth nystagmus and reduced vision	02	Night blindness, photophobia, nystagmus, congenital blindness	*LCA5*(NM_001122769): c.1151del, p.(Pro384GlnfsTer18) Homozygous
	IV:4/Female (Proband)	25	arRD	arLCA	By birth nystagmus and reduced vision	02	Night blindness, photophobia, nystagmus, congenital blindness	*LCA5*(NM_001122769): c.1151del, p.(Pro384GlnfsTer18) Homozygous
MA0461	IV:2/Female (Proband)	22	arLCA	arLCA	By birth blindness, nystagmus	05	Complete blindness since 2 years of age	*GUCY2D*(NM_000180):c.3056A>C, p.His1019Pro Homozygous
	IV:3/Female	18	arLCA	arLCA	By birth blindness	05	Complete blindness since 2 years of age	*GUCY2D*(NM_000180):c.3056A>C, p.His1019Pro Homozygous
MA0422	III:2/Male (Proband)	45	Pigmentary retinopathy	arRP	Low vision since 7 years of age	02	Night blindness at 9 years of age, complete blindness at 12 years of age	*CHM*(NM_000390):c.1584_1587del, p.(Val529HisfsTer7) Homozygous
	III:5/Male		Pigmentary retinopathy	arRP	Low vision since 6 years of age	02	Night blindness at 9 years of age, complete blindness at 7 years of age	*CHM*(NM_000390):c.1584_1587del, p.(Val529HisfsTer7) Homozygous
MA0534	IV:14/Male	22	Usher syndrome	Usher syndrome	Sensorineural hearing loss and low vision	02	RP and Hearing problem	*MY07A* (NM_001369365.1):c.55962A>G Homozygous
	IV:15	23	Usher syndrome	Usher syndrome	By birth night blindness, RP	02	Sensorineural hearing loss, low vision and bilateral cataracts	*MY07A* (NM_001369365.1):c.55962A>G Homozygous

**Table 3:** Summary of genetic findings and population distribution of variants identified in 43 out of 50 IRDs, anophthalmia and congenital cataracts manifesting pedigrees.

Pakistani Population	
Summary of genetic findings and population distribution of variants identified in 43 pedigrees	43
Mutation identification rate	86%
Novel IRD detected mutations	16
Novel SNVs not reported in gnomAD	06

## Data Availability

The raw data supporting the conclusions of this article will be made available by the authors on request.

## Supplementary Materials

The following supporting information can be downloaded at https://www.mdpi.com/article/10.3390/ijms26062715/s1. References [48,49,50,51,52,53,54,55,56,57,58,59,60,61,62,63,64,65] are cited in the Supplementary Materials.
