# Supplementary material for: Deciphering the Genetic Basis of Degenerative and Developmental Eye Disorders in 50 Pakistani Consanguineous Families Using Whole-Exome Sequencing
Source: Int J Mol Sci. 2025 Mar 18;26(6):2715. doi: 10.3390/ijms26062715 (PMC11942243; doi:10.3390/ijms26062715)
Supplement: Supplementary file 1 [file ijms-26-02715-s001.zip › Supplementary Figures.pdf]

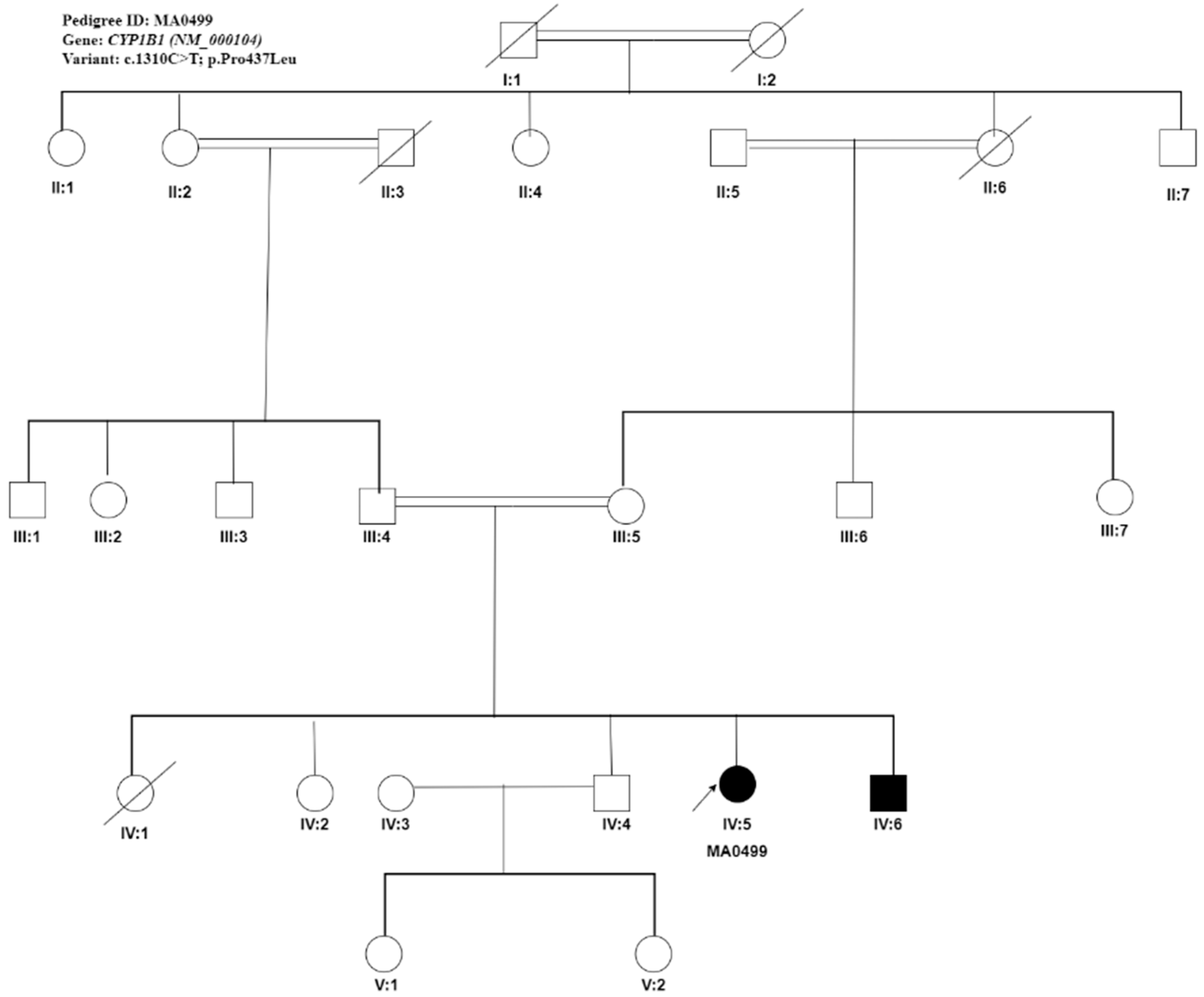

Figure S1: A Punjabi pedigree RF. MA0499 seggregating retinal degeneration and glucoma in an autosomal recessive pattern of inheritance. WES identified a reported missense variant, c.1310C>T, p. (Pro437Leu) in *CYP1B1* (NM_ 000104) gene.

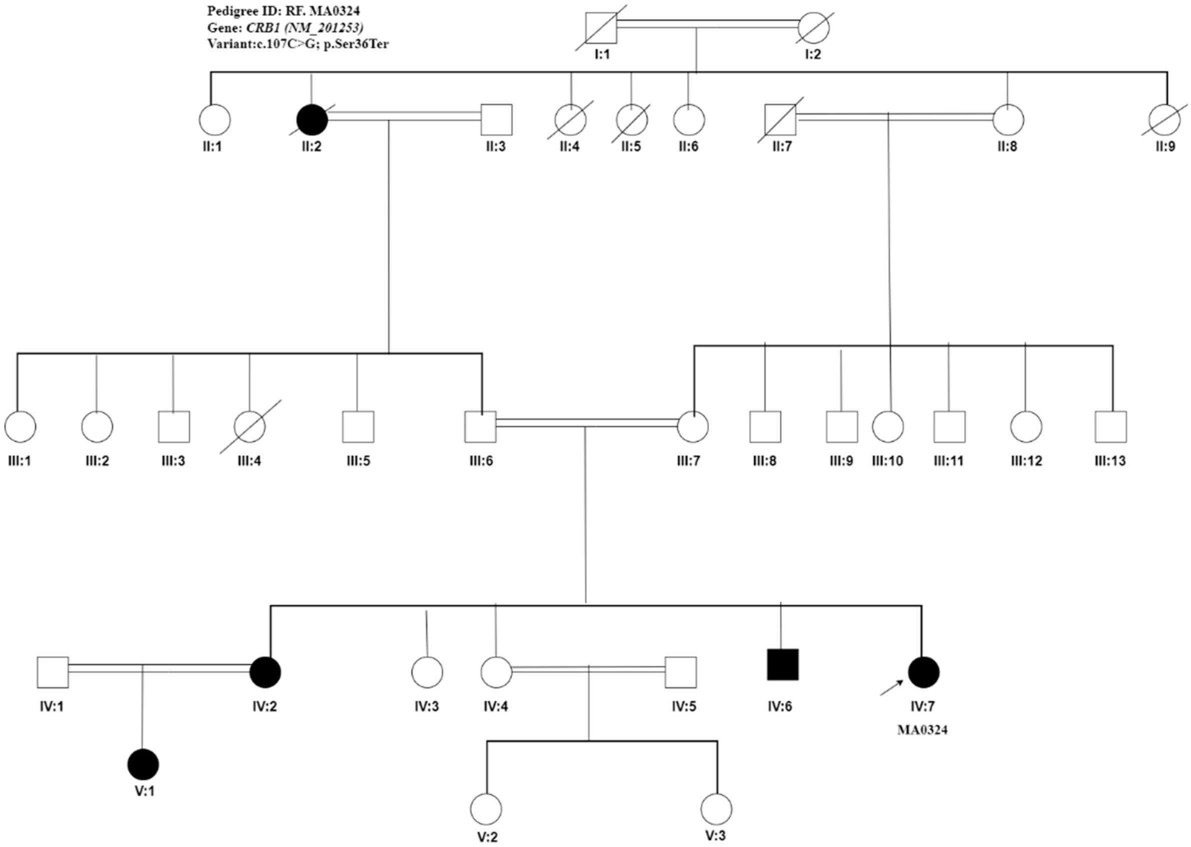

Figure S2. Pedigree RF.MA0324 seggregating RP in an autosomal recessive manner was harboring a reported homozygous nonsense stop gain potentially damaging variant c.107C>G; p. Ser36Ter in *CRB1* (NM_ 201253) gene.

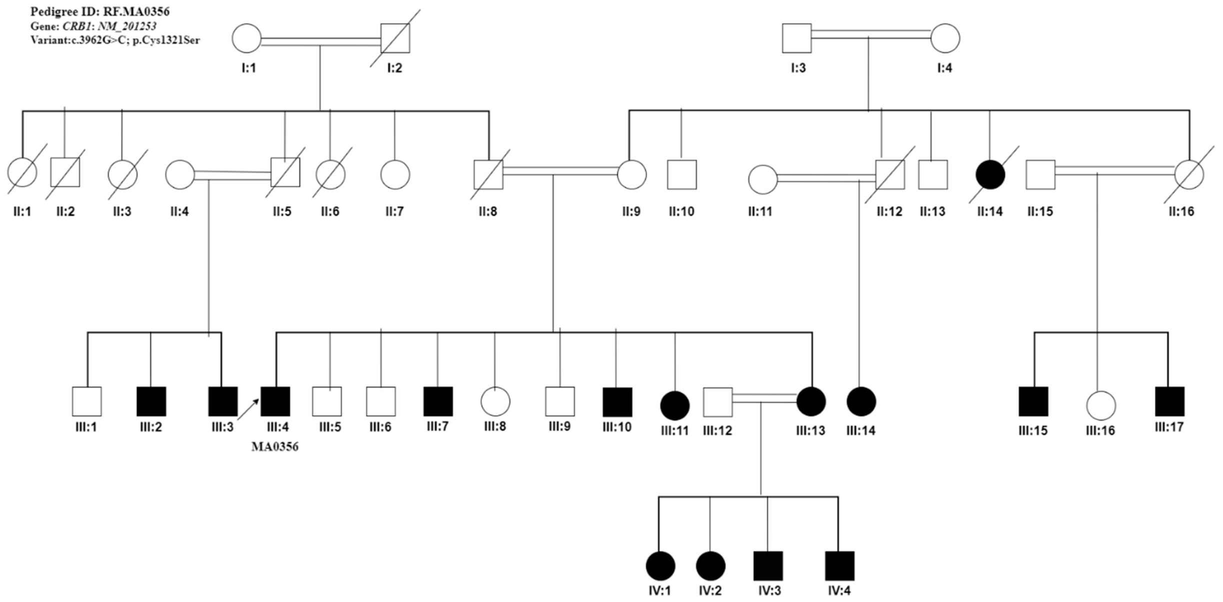

Figure S3. A large consanguineous four-generation Punjabi pedigree RF.MA0356 with two affected males with a clinical diagnosis of RP. WES identified a homozygous reported pathogenic variant c.3962G>C, p. Cys1321Ser in exon 11 of *CRB1* (NM_ 201253) gene

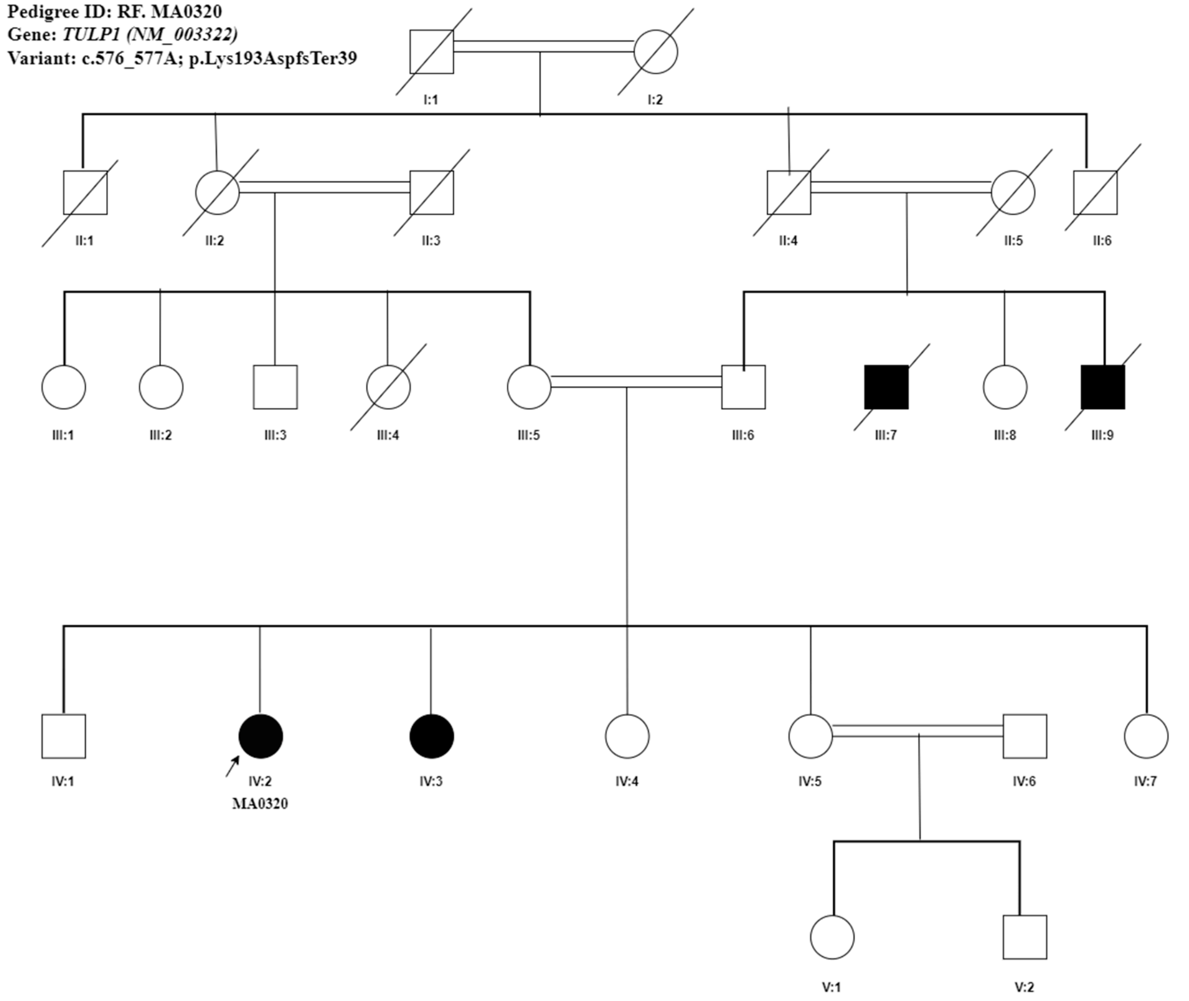

Figure S4. Pedigree of a Punjabi family RF. MA0320 seggregating RP in an autosomal recessive pattern of inheritance. WES identified a novel frameshift deletion c.576_ 577A, p. Lys193AspfsTer39 in *TULP1* (NM_ 003322) gene in this pedigree.

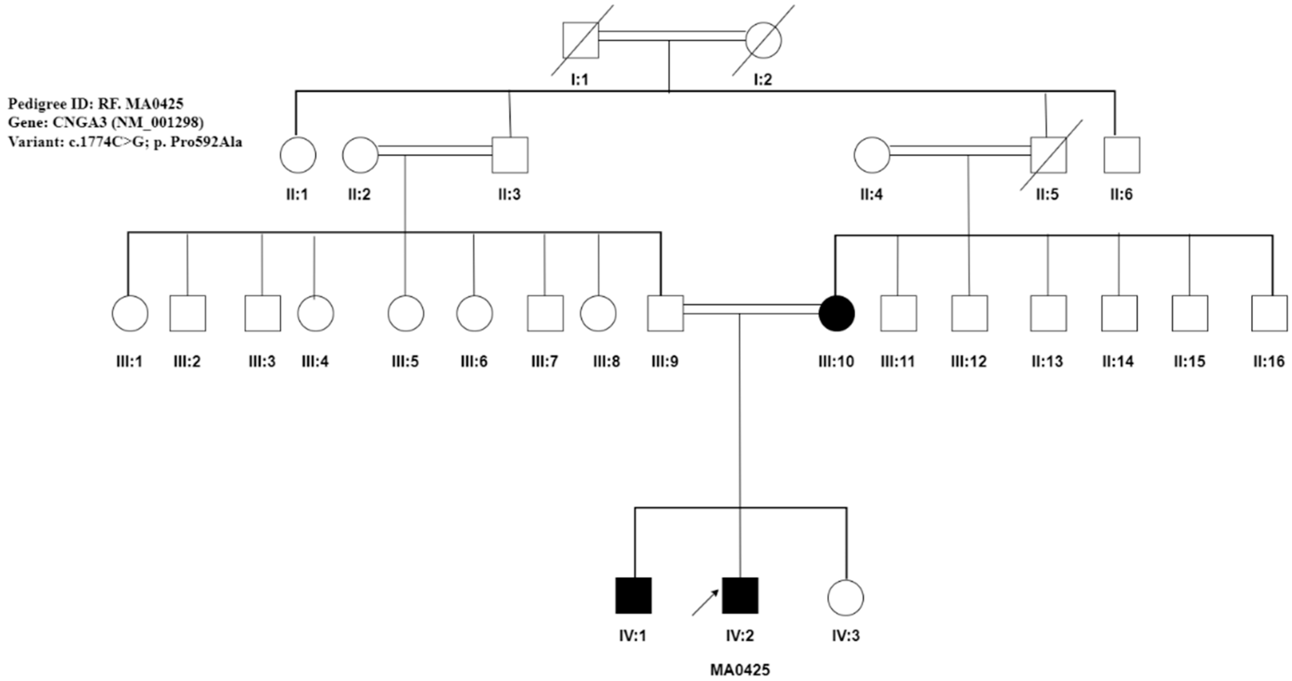

Figure S5. Pedigree of a Punjabi family RF. MA0425 seggregating achromatopsia in an autosomal recessive pattern of inheritance. WES identified a novel *CNGA3* (NM_ 001298) gene (MIM# 600053) variant, c.1774 C>G, p. Pro592Ala in this pedigree.

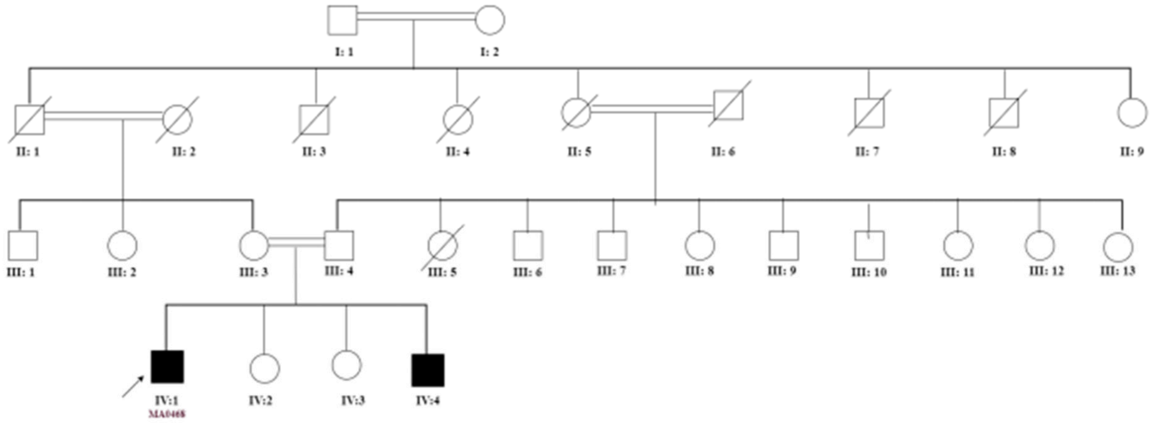

COL18A1: **c.3558_3577; p. Gly1010AlafsTer70**

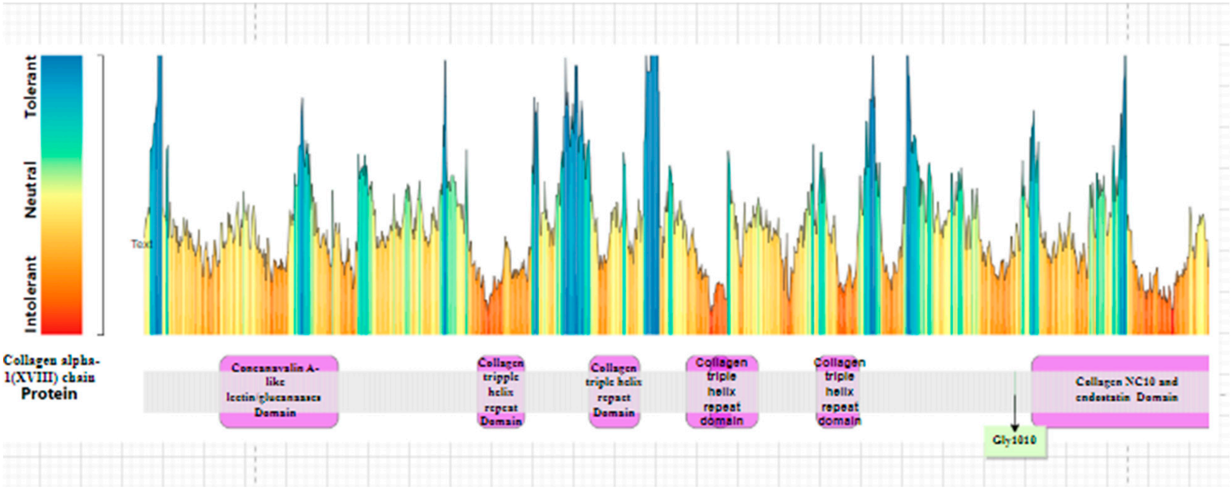

Figure S6. (a)A 20 base pairs (bps) novel homozygous frameshift deletion in the *COL18A1* gene (Chr21: g. 45505371_ 5505390del) was identified in a large consanguineous Punjabi family RF. MA0468. (b) MetaDome health map represents COL18A1 residues.

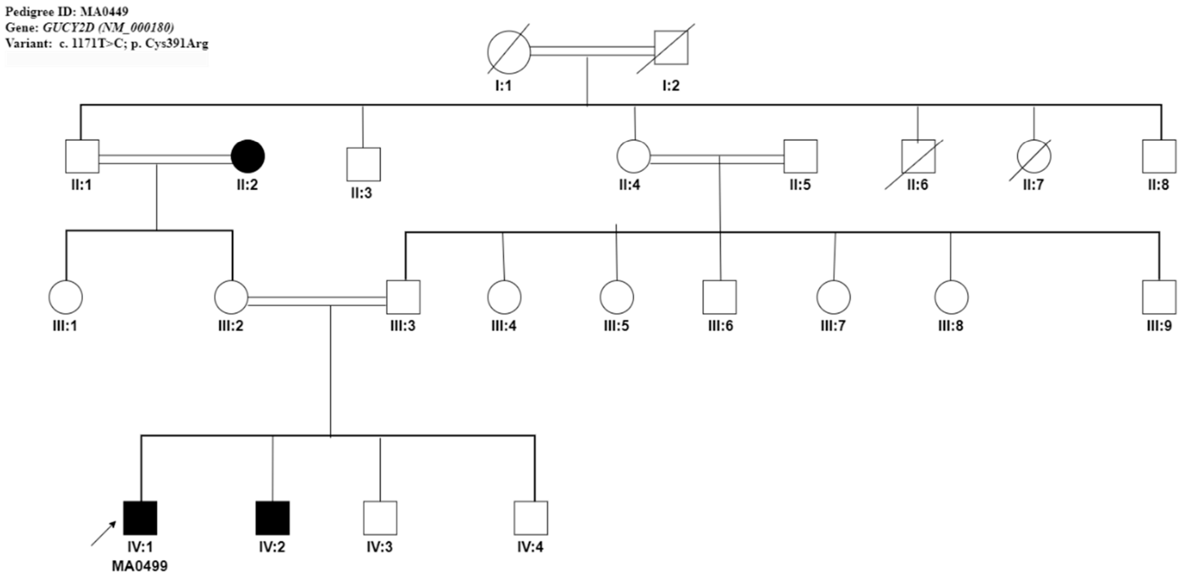

Figure S7. RF. MA0449 Punjabi pedigree with two affected members. WES identified a pathogenic novel homozygous variant c.1171T>C, p. Cys391Arg in exon 4 of *GUCY2D* (NM_ 000180) gene associated with Leber congenital amaurosis.

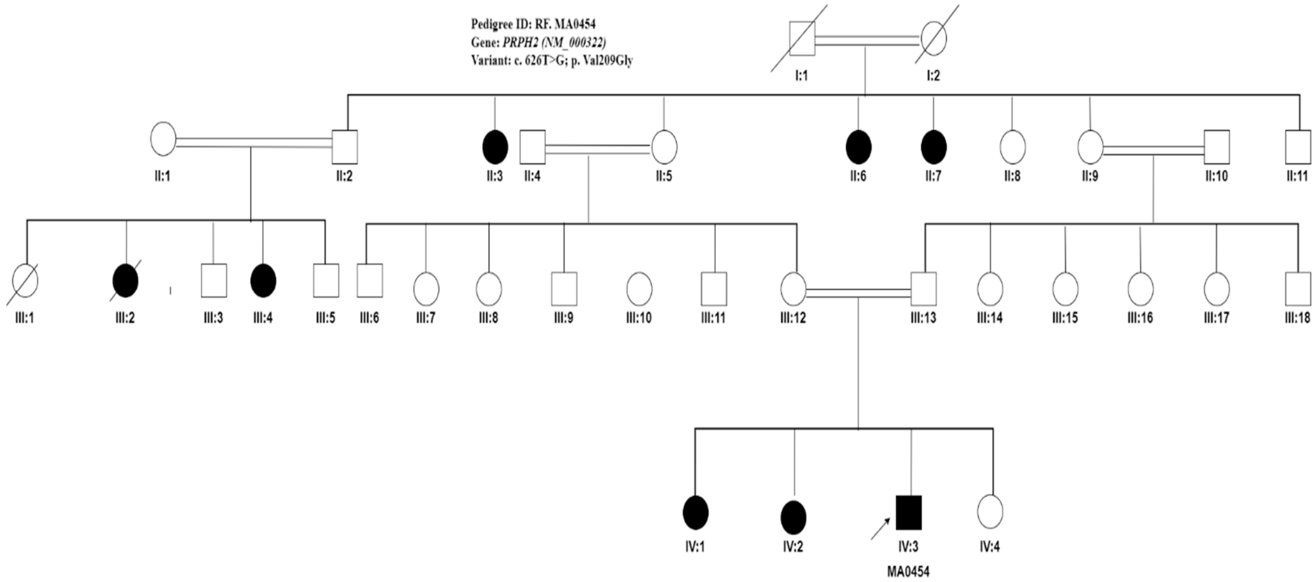

Figure S8. A Punjabi Pedigree RF.MA0454 seggregating LCA in an autosomal recessive manner. WES discovered a novel homozygous missense substitution c.626T>G, p. Val209Gly in the *PRPH2* (NM_ 000322) gene.
