# Supplementary material for: Deciphering the Genetic Basis of Degenerative and Developmental Eye Disorders in 50 Pakistani Consanguineous Families Using Whole-Exome Sequencing
Source: Int J Mol Sci. 2025 Mar 18;26(6):2715. doi: 10.3390/ijms26062715 (PMC11942243; doi:10.3390/ijms26062715)
Supplement: Supplementary file 1 [file ijms-26-02715-s001.zip › Table S1.pdf]

Table S1: Summary of Primers

Patient ID	Gene	Forward Primer	Reverse Primer	Product (bp)	Tm(ºC)
**MA0334**	CNGA1	AGAGCAGAAATTGCCATCAACG	TGCCAGCTTTGCTCCCTTTA	292	60
**MA0362**	LYST	GCCTTGTCTTGCCTTCTCCT	GTTCTGATGGTATGGGGTCACT	312	60
**MA0406**	CRB1	TTCTCCTGGGCTGTACCCAT	TCCACGAAATGCCACTCTCC	391	60
**MA0468**	COL18A1	CCGTCCGTAGGGTCCCAAG	GTCCGTGGGAGAGTGTCTCTG	398	62
**MA0477**	BLOC1S3	TCCTGCAACTTCGGCTGG	AGGAGACAGAGTCAGGTCCC	362	60
**MA0503**	CRB1	CATGACAGACCCACTGTCCC	TTCCTCCATGCAAACAGGGG	338	60
**MA0510**	GUCY2D	GGGAGTGGGGCTTACCTTGA	GGACTCACCTCCCGTGTC	306	60
**MA0524**	LONP1	GGCCCTGGTTTCACCATCA	GATGGGCATGGAAAGGTGGG	359	60
**MA0537**	COL9A1	CAAGCCTCCTGCTTTTCAGTG	AGCCCCACTCACTCTTGGAG	389	60
