# Supplementary material for: Deciphering the Genetic Basis of Degenerative and Developmental Eye Disorders in 50 Pakistani Consanguineous Families Using Whole-Exome Sequencing
Source: Int J Mol Sci. 2025 Mar 18;26(6):2715. doi: 10.3390/ijms26062715 (PMC11942243; doi:10.3390/ijms26062715)
Supplement: Supplementary file 1 [file ijms-26-02715-s001.zip › Table S2.pdf]

Table S2. A list of causative variants identified in each family during this study along with their pathogenicity predictions.

Pedigree ID	Phenotype	Gene	c. DNA	Protein Change	Intervar_ 20180118	CADD_ PHRED	GERP++ _NR	GNOMAD FQ	Reference
MA0315	ar LCA	*LCA5 (NM_001122769)*	c.1151del	p.(Pro384GlnfsTer18)	-	-	-	-	[14]
MA0320	arRP	*TULP1 (NM_ 001289395.2)*	c.417del	p. (Lys140Arg fsTer2)	-	-	-	-	This study
MA0324	arRP	*CRB1 (NM_201253)*	c.107C>G	p.(Ser36Ter)	Pathogenic	36	5.52	-	[48]
MA0328	arRP	*CERKL(NM_201548)*	c.967_ 968del	p.(Met323ValfsTer20)	-	-	-	-	[49]
MA0334	arRP	*CNGA1 (NM_000087)*	c.1270C>T	p.(Arg424Ter)	Uncertain Significance	39	5.31	0.00002644599	This study
MA0337<br>MA0371	arRP	*CERKL (NM_201548)*	c.769C>T	p.(Arg257Ter)	Pathogenic	26.4	5.93	0.0003173	[50]
MA0341<br>MA0503	arRP<br>arLCA	*CRB1 (NM_ 001193640.2)*	c.3007_ 3016del	p.(Gly1003Ile) fsTer23	-	-	-	-	[51]
MA0353	arRP	*RPE65 (NM_000329)*	c. 74C>T	(p.Pro25Leu)	Uncertain significance	25.7	5.33	0.0000037	[52]
MA0356	arRP	*CRB1 (NM_ 001193640.2)*	c.3626G>C >C	p.(Cys1209Ser)	Uncertain significance	26.2	5.36	-	Variant not published
MA0366	arRP	*AIPL1 (NM_014336)*	c.834G>A	p.(Trp278Ter)	Uncertain significance	44	5.14	0.0004155	[52]
MA0380	arRP	*CRB1 (NM_201253)*	c.601T>C	p.(Cys201Arg)	Uncertain significance	24.6	5.53	-	Variant not Published
MA0383	arMC	*CLCN1 (NM_000083)*	c.2647C>A	p.(Pro883Thr)	Uncertain significance	24.5	4.52	-	[53]
MA0392<br>MA0435	arLCA	*AIPL1 (NM_014336)*	c.834G>A	p.(Trp278Ter)	Uncertain significance	44	5.14	0.0004155	[54]
MA0396	arRP	*RPGRIP1 (NM_020366)*	c.2480G>T	p.(Arg827Leu)	Likely pathogenic	24.6	4.71	-	[55]
MA0400	arRP	*LRP5 (NM_002335)*	c.2173G>A	p.(Val725Ile)	Uncertain significance	23.9	4.41	-	Variant not published
MA0406	arRP	*CRB1 (NM_201253)*	c.1459T>C	p.(Ser487Pro)	Uncertain significance	24.2	5.82	-	[20]
MA0410	arRP	*EYS (NM_001142800)*	c.5928-2A>T	N/A	-	33	4.73	-	[56]
MA0413<br>MA0430	arRP	*PDE6A (NM_000440)*	c.650_ 651dup	p.(Ala218LeufsTer4)	-	-	-	-	This study
MA0422	RP	*CHM (NM_000390)*	c.1584_ 1587del	p.(Val529HisfsTer7)	-	-	-	-	[57]

Table 2. *Cont.*

Pedigree ID	Phenotype	Gene	c. DNA	Protein Change	Intervar_ 20180118	CADD_ PHRED	GERP++ _NR	GNOMAD FQ	Reference
MA0425	arACHM	*CNGA3 (NM_001298)*	c.1774C>G	p. (Pro592Ala)	Uncertain significance	24.2	5.42	-	This study
MA0443	arRP	*RLBP1 (NM_000326)*	c.256G>T	p.(Glu86Ter)	Uncertain significance	36	5.03	-	This study
MA0449	arLCA	*GUCY2D (NM_000180)*	c.1171T>C	p.(Cys391Arg)	Uncertain significance	25.8	4.95	-	This study
MA0454	arLCA	*PRPH2 (NM_000322*	c.626T>G	p.Val209Gly	Uncertain significance	29.4	5.1	-	This study
MA0461	arLCA	*GUCY2D (NM_000180)*	c.3056A>C	p.(His1019Pro)	Uncertain significance	32	4.22	-	[58]
MA0468	arKNO1	*COL18A1 (NM_030582.4)*	c.3559_3577del	p. (Ser1187Ala fsTer18)	-	-	-	0.000022668	This study
MA0472	arCRD	*GUCY2D (NM_000180)*	c.3138+1G>A	N/A	-	35	4.21	-	This study
MA0477	arHPS-8	*BLOC1S3 (NM_ 212550)*	c.499C>T	p.(Leu167Phe)	Uncertain significance	25.2	3.61	0.000015	Variant not published
MA0484	arSTGD	*ABCA4 (NM_000350)*	c.5196+1G>A	N/A	-	33	5.38	0.000015	[59]
MA0489	arCC.OAGS	*FOXE3 (NM_012186)*	c.720C>A	p.(Cys240Ter)	Uncertain significance	36	3.09	-	[60]
MA0494	arRA	*GALK1 (NM_000154)*	c.593C>T	p.(Ala198Val)	Uncertain significance	24.3	5.53	0.00073	[61]
MA0499	arRDEG+GLU	*CYP1B1 (NM_000104)*	c.1310C>T	p.(Pro437Leu)	Uncertain significance	25.9	5.95	0.0000793	[62]
MA0507	arLCA	*AIPL1 (NM_014336)*	c.664T>C	p.(Trp222Arg)	Uncertain Significance	28.2	5.15	-	This Study
MA0510	arCRD	*GUCY2D (NM_000180)*	c.2965dup	p.(Val989Gly fsTer83)	-	-	-	-	This study
MA0515	arCG	*CYP1B1 (NM_000104)*	c.1063C>T	p. (Arg355Ter)	-	47	5.65	0.0000151	[63]
MA0524	arCODAS	*LONP1 (NM_001276480)*	c.1448G>A	p. (Arg483His)	Uncertain significance	22.5	3,72	0.00006800	[64]
MA0530	ar Anophth	*FOXE3 (NM_012186)*	c.289A>G	p.(Ile97Val)	Uncertain significance	24.9	3.24	-	[59]
MA0534	ar USH	*MYO7A (NM_000260)*	c.5743-2A>G	N/A	-	35	5.01	-	[65]
MA0537	ar STL Type-IV	*COL9A1 (NM_078485.4)*	c.851_852insCAAT	p.(Pro285Asn fsTer20)	-	-	-	-	This study

References

14. Li, L.; Chen, Y.; Jiao, X.; Jin, C.; Jiang, D.; Tanwar, M.; Ma, Z.; Huang, L.; Ma, X.; Sun, W.; et al. Homozygosity mapping and genetic analysis of autosomal recessive retinal dystrophies in 144 consanguineous Pakistani families. Investig. Ophthalmol. Vis. Sci. 2017, 58, 2218–2238.

20. Ur Rehman, A.; Peter, V.; Quinodoz, M.; Rashid, A.; Khan, S.; Superti-Furga, A.; Rivolta, C. Exploring the genetic landscape of retinal diseases in North-Western Pakistan reveals a high degree of autozygosity and a prevalent founder mutation in ABCA4. Genes 2019, 11, 12.

48. McKibbin, M., Ali, M., Mohamed, M., Booth, A., Bishop, F., Pal, B., Springell, K., Raashid, Y., Jafri, H. & Inglehearn, C. Genotype-phenotype correlation for leber congenital amaurosis in Northern Pakistan. Archives Of Ophthalmology. 128, 107-113 (2010)

49. Wang, X.; Zein, W.; D'Souza, L.; Roberson, C.; Wetherby, K.; He, H.; Villarta, A.; Turriff, A.; Johnson, K.; Fann, Y. Applying next generation sequencing with microdroplet PCR to determine the disease-causing mutations in retinal dystrophies. *BMC Ophthalmol.* **2017**, *17*, 157.

50. Carss, K.; Arno, G.; Erwood, M.; Stephens, J.; Sanchis-Juan, A.; Hull, S.; Megy, K.; Grozeva, D.; Dewhurst, E.; Malka, S.; et al. Comprehensive rare variant analysis via whole-genome sequencing to determine the molecular pathology of inherited retinal disease. *Am. J. Hum. Genet.* **2017**, *100*, 75–90.

51. Lotery, A., Malik, A., Shami, S., Sindhi, M., Chohan, B., Maqbool, C., Moore, P., Denton, M. & Stone, E. CRB1 mutations may result in retinitis pigmentosa without para- arteriolar RPE preservation. *Ophthalmic Genetics*. **22**, 163-169 (2001)

52. Zampaglione, E.; Maher, M.; Place, E.; Wagner, N.; DiTroia, S.; Chao, K.; England, E.; Broad, C.; Catomeris, A.; Nassiri, S.; et al. The importance of automation in genetic diagnosis: Lessons from analyzing an inherited retinal degeneration cohort with the Mendelian Analysis Toolkit (MATK). *Genet. Med.* **2022**, *24*, 332–343.

53. Suetterlin, K.; Matthews, E.; Sud, R.; McCall, S.; Fialho, D.; Burge, J.; Jayaseelan, D.; Haworth, A.; Sweeney, M.; Kullmann, D.; et al. Translating genetic and functional data into clinical practice: A series of 223 families with myotonia. *Brain* **2022**, *145*, 607–620.

54. Sohocki, M.; Perrault, I.; Leroy, B.; Payne, A.; Dharmaraj, S.; Bhattacharya, S.; Kaplan, J.; Maumenee, I.; Koenekoop, R.; Meire, F.;et al. Prevalence of AIPL1 mutations in inherited retinal degenerative disease. *Mol. Genet. Metab.* **2000**, *70*, 142–150.

55. Bianco, L.; Antropoli, A.; Arrigo, A.; Saladino, A.; Berni, A.; Bandello, F.; Mansour, A.; Parodi, M. RPGRIP1 variant associated with pigmented paravenous chorioretinal atrophy. *Eur. J. Ophthalmol.* **2023**, *33*, NP6–NP9.

56. McGuigan, D.; Heon, E.; Cideciyan, A.; Ratnapriya, R.; Lu, M.; Sumaroka, A.; Roman, A.; Batmanabane, V.; Garafalo, A.; Stone, E.; et al. EYS mutations causing autosomal recessive retinitis pigmentosa: Changes of retinal structure and function with disease progression. *Genes* **2017**, *8*, 178

57. McLaren, T., De Roach, J., Thompson, J., Chen, F., Mackey, D., Hoffmann, L., Urwin, I. & Lamey, T. Expanding the genetic spectrum of choroideremia in an Australian cohort: report of five novel CHM variants. *Human Genome Variation*. **7**, 35 (2020)

58. Bouzia, Z.; Georgiou, M.; Hull, S.; Robson, A.; Fujinami, K.; Rotsos, T.; Pontikos, N.; Arno, G.; Webster, A.; Hardcastle, A.; et al. GUCY2D-associated Leber congenital amaurosis: A retrospective natural history study in preparation for trials of novel therapies. *Am. J. Ophthalmol.* **2020**, *210*, 59–70.

59. Schulz, H.; Grassmann, F.; Kellner, U.; Spital, G.; Rüther, K.; Jägle, H.; Hufendiek, K.; Huchzermeyer, C.; Baier, M.; Weber, B.; et al. Mutation spectrum of the ABCA4 gene in 335 Stargardt disease patients from a multicenter German cohort—Impact of selected deep intronic variants and common SNPs. *Investig. Ophthalmol. Vis. Sci.* **2017**, *58*, 394–403.

60. Reis, L.; Sorokina, E.; Dudakova, L.; Moravikova, J.; Skalicka, P.; Malinka, F.; Seese, S.; Thompson, S.; Bardakjian, T.; Capasso, J. ; et al. Comprehensive phenotypic and functional analysis of dominant and recessive FOXE3 alleles in ocular developmental disorders. *Hum. Mol. Genet.* **2021**, *30*, 1591–1606.

61. Timson, D.; Reece, R. Functional analysis of disease-causing mutations in human galactokinase. *Eur. J. Biochem.* **2003**, *270*, 1767–1774.

62. Rashid, M.; Yousaf, S.; Sheikh, S.; Sajid, Z.; Shabbir, A.; Kausar, T.; Tariq, N.; Usman, M.; Shaikh, R.; Ali, M.; et al. Identities and frequencies of variants in CYP1B1 causing primary congenital glaucoma in Pakistan. *Mol. Vis.* **2019**, *25*, 144–154.

63. .Gupta, V.; Panigrahi, A.; Mahalingam, K.; Singh, A.; Somarajan, B.; Gupta, S. Expanding the phenotypic spectrum of CYP1B1 associated primary congenital glaucoma. *Clin. Exp. Ophthalmol.* **2022**, *50*, 1112.

64. Dikoglu, E.; Alfaiz, A.; Gorna, M.; Bertola, D.; Chae, J.; Cho, T.; Derbent, M.; Alanay, Y.; Guran, T.; Kim, O.; et al. Mutations in LONP1, a mitochondrial matrix protease, cause CODAS syndrome. *Am. J. Med. Genet. Part A* **2015**, *167*, 1501–1509.

65. Aparisi, M.; Aller, E.; Fuster-García, C.; García-García, G.; Rodrigo, R.; Vázquez-Manrique, R.; Blanco-Kelly, F.; Ayuso, C.; Roux, A.; Jaijo, T.; et al. Targeted next generation sequencing for molecular diagnosis of Usher syndrome. *Orphanet J. Rare Dis.* **2014**, *9*, 168.
